# DSG2+ Cancer Stem Cells Co‐Located With FAP+ Myofibroblasts in the Tumor Boundary That Determines the Efficacy of Immunotherapy in Non‐Small Cell Lung Cancer

**DOI:** 10.1002/advs.202514543

**Published:** 2026-02-15

**Authors:** Guangyu Fan, Le Tang, Tongji Xie, Mengwei Yang, Lin Li, Sheng Yang, Puyuan Xing, Xiaohong Han, Yuankai Shi

**Affiliations:** ^1^ Department of Medical Oncology Beijing Key Laboratory of Key Technologies For Early Clinical Trial Evaluation of Innovative Drugs For Major Diseases National Cancer Center /National Clinical Research Center for Cancer/ Cancer Hospital Chinese Academy of Medical Sciences & Peking Union Medical College Beijing China; ^2^ Department of Pathology National Cancer Center / National Clinical Research Center For Cancer / Cancer Hospital Chinese Academy of Medical Sciences & Peking Union Medical College Beijing China; ^3^ Clinical Pharmacology Research Center State Key Laboratory of Complex Severe and Rare Diseases NMPA Key Laboratory For Clinical Research and Evaluation of Drug Peking Union Medical College Hospital Beijing Key Laboratory of Key Technologies for Early Clinical Trial Evaluation of Innovative Drugs for Major Diseases Chinese Academy of Medical Sciences & Peking Union Medical College Beijing China

**Keywords:** cancer stem cells, EGFR‐TKI therapy, immunotherapy, lung cancer, single‐cell RNA sequencing, spatial transcriptomics

## Abstract

Lung cancer remains a leading cause of cancer‐related mortality owing to its aggressiveness and pronounced heterogeneity. Cancer stem cells (CSCs) have been implicated in tumor progression and therapeutic resistance; however, their transcriptional programs and spatial organization in non‐small cell lung cancer (NSCLC) are not fully characterized. Here, we integrated single‐cell RNA sequencing with spatial transcriptomics to systematically define CSC‐associated phenotype in NSCLC. Weighted gene co‐expression network analysis identified a 127‐gene CSC signature enriched for stemness‐related pathways, with DSG2 emerging as a dominant marker. Elevated DSG2 expression was associated with chemotherapy resistance and predicted response to EGFR tyrosine kinase inhibitors based on independent clinical proteomic datasets. Spatial mapping revealed preferential enrichment of DSG2+CSCs at tumor margins, where they co‐localized with FAP+myofibroblasts (myCAFs). Multiplex immunofluorescence demonstrated that FAP+myCAFs expressed MMP9 and MMP12, consistent with a microenvironment supportive of epithelial‐mesenchymal transition and CSC maintenance. Functional co‐culture assays showed that myCAFs enhanced CSC‐associated phenotypes in DSG2^high tumor cells in an MMP‐dependent manner. Collectively, these findings delineate a spatially organized DSG2+CSC–myCAF niche that contributes to therapeutic resistance in NSCLC.

## Graphical abstract

Cancer stem cells (CSCs) in non‐small cell lung cancer display pronounced plasticity and spatial heterogeneity. By integrating single‐cell and spatial transcriptomics, this study defines a DSG2‐associated CSC program and reveals a tumor‐margin niche formed with FAP^+^ myofibroblasts. This spatially organized interaction promotes CSC maintenance and therapeutic resistance.

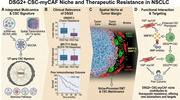

## 1 Introduction

Lung cancer remains one of the most lethal malignancies worldwide, characterized by pronounced biological heterogeneity, rapid progression, and poor long‐term outcomes [1, 2]. Non‐small cell lung cancer (NSCLC) constitutes the majority of cases [3], yet survival remains unsatisfactory despite advances in targeted therapy, immunotherapy, and early detection strategies. Across all stages, the 5‐year survival rate is approximately 25% and falls below 10% in metastatic disease [4]. Even patients undergoing curative‐intent surgery frequently relapse, and although many individuals initially respond to molecularly targeted agents or immune checkpoint blockade, durable benefit is limited by the near‐universal emergence of therapeutic resistance [5, 6, 7].

Increasing evidence suggests that these clinical challenges are closely linked to tumor cell plasticity and microenvironmental influences [8, 9]. Cancer stem cells (CSCs) have been proposed as a key driver of tumor initiation, progression, and recurrence [10, 11]. Traditionally, tumors were viewed as hierarchically organized, with a rare stem‐like population capable of self‐renewal and differentiation. More recently, however, the phenotype plasticity model has reframed CSCs as a dynamic state rather than a fixed subset, in which cancer cells can reversibly transition between stem‐like and non‐stem‐like phenotypes [12, 13]. This flexibility enables tumor cells to regenerate stem‐like populations after therapy and contributes to disease persistence [14].

Most prior studies have relied on surface markers and functional assays, such as cell sorting and xenotransplantation, to identify CSCs [15–17]. Although markers including CD13, CD24, EPCAM, CD44, and CD133 have been widely used, they are not tumor‐specific and provide limited insight into the transcriptional continuum underlying stemness [18, 19]. Consequently, the molecular programs that define CSC states and their spatial organization within tumors remain incompletely characterized.

CSCs also actively remodel the tumor microenvironment (TME) to promote survival [20]. By suppressing cytotoxic immune responses, expressing immune checkpoint molecules, and enhancing angiogenesis and tissue remodeling, CSCs foster an immunosuppressive niche that protects them from therapy [21]. In turn, stromal and immune components of the TME reinforce stem‐like traits, creating reciprocal interactions that sustain resistance to both targeted therapy and immunotherapy [22, 23]. Dissecting this bidirectional crosstalk is therefore essential for improving treatment efficacy. Spatial transcriptomics provides an opportunity to address these gaps by simultaneously resolving gene expression and tissue architecture. This approach enables precise mapping of CSC‐enriched regions, identification of neighboring stromal or immune populations, and characterization of cell–cell interactions within defined niches [10]. Leveraging Spatial transcriptomics, together with single‐cell analyses, allows a more comprehensive understanding of how CSCs are organized and supported within the lung cancer microenvironment [24].

We hypothesized that CSCs in NSCLC display distinct transcriptional programs and spatial organization that drive tumor progression and therapeutic resistance. To investigate this, we integrated single‐cell RNA sequencing from 92 820 cells across 40 tumors with spatial transcriptomics from 28,712 spots across six samples. This approach enabled the construction of a CSC gene signature, mapping of CSC localization within the tumor microenvironment, and assessment of associations between CSC markers and treatment response, including chemotherapy, targeted therapy, and immunotherapy. Together, these analyses define the molecular and spatial features of CSCs and clarify their contribution to disease progression and therapy resistance.

## 2 Results

### 2.1 The Signature of CSC Was Identified Using Weighted Gene Co‐Expression Network Analysis

**FIGURE 1:**
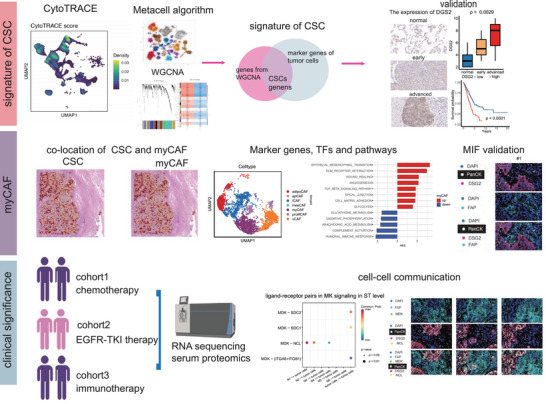
The study design workflow is depicted.

The workflow was depicted in Figure 1. Single‐cell RNA sequencing data from 42 NSCLC tumors in GSE148071 (107,761 cells) were analyzed to define stem‐like tumor populations [25]. Clustering resolved six major cell types, including epithelial, myeloid, fibroblast, endothelial, T, and B cells. We utilized a rigorous approach involving the integration of weighted gene co‐expression network analysis (WGCNA) and the Metacell algorithm [26, 27].

**FIGURE 2:**
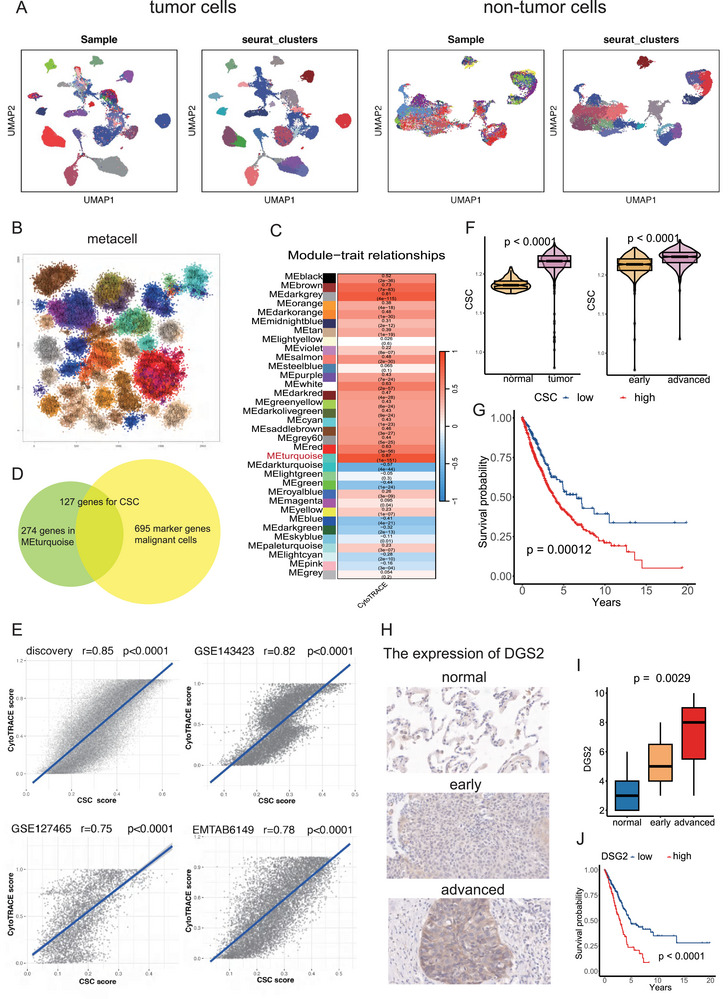
Identification of the cancer stem cells (CSC) signature using weighted gene co‐expression network analysis (WGCNA). (A) Uniform manifold approximation and projection (UMAP) plots showing the grouping of malignant cells (left) and non‐tumour cells (right) based on sample origins and seurat clusters, respectively. (B) Partitioning of 50 275 malignant cells into 498 metacells using the Metacell algorithm. Each metacell is assigned a unique numerical identifier, and metacells with similar transcriptional profiles are spatially grouped and visualized using the same color for clarity. Colors are used for visualization only and do not define analytical groupings. (C) Heatmap displaying the correlation between each gene expression module and the CytoTRACE score. The y‐axis represents distinct gene expression modules, with module identities indicated by the color annotation bar, while the x‐axis indicates the CytoTRACE score. The first number in each cell denotes the correlation coefficient, and the second indicates the corresponding p‐value. (D) Identification of 127 CSC‐related genes as the signature of CSC by intersecting the marker genes of malignant cells with the genes in the MEturquoise module. (E) Scatter plots showing the correlation between the identified CSC signature and CytoTRACE score n our discovery data GSE148071 and three additional single‐cell datasets of NSCLC (GSE127465, GSE143423, and EMTAB6149). (F) Distribution of CSC score in normal and tumor tissues (left) and in early and advanced stage NSCLC tissues (right). (G) NSCLC patients in the TCGA cohort with higher CSC infiltration exhibited shorter overall survival (OS). (H) Immunohistochemistry images of DSG2 identified in the CSC signature in our cohort consisting of 70 NSCLC patients. (I) Boxplots displaying the distribution of the DSG2 in normal, early and advanced stage samples. (J) NSCLC patients in the TCGA cohort with higher DSG2 expression exhibited shorter OS.

Single‐cell RNA sequencing data is inherently sparse and noisy, which can mask true gene‐gene correlations and confound the analysis of co‐expression networks. Additionally, we observed considerable variation among malignant cells, both within individual patients and between different patients. While malignant cells displayed clear groupings based on their sample source, non‐malignant cells showed limited noticeable differences among individuals (Figure 2A). This observation indicates that the heterogeneity of tumor cells is primarily due to the tumor cells themselves rather than the samples. The heterogeneity among cancer cells in our dataset necessitated modifying the traditional WGCNA workflow commonly applied to bulk transcriptomic data. We employed the Metacell algorithm to partition cells into homogenous groups. After quality control, a total of 50 275 malignant cells were selected and divided into 498 metacells (Figure 2B).

Because single‐cell data are sparse and heterogeneous, direct co‐expression analysis can be unreliable. We also observed substantial transcriptional variability among malignant cells both within and across patients, whereas non‐malignant populations showed relatively limited inter‐individual differences (Figure 2A). To address this heterogeneity and improve signal stability, malignant cells were aggregated into transcriptionally similar metacells. After quality control, 50,275 tumor cells were grouped into 498 metacells (Figure 2B), providing a refined representation of gene expression patterns.

Co‐expression networks were then constructed using the 5 000 most variable genes across metacells. A soft‐thresholding power of 7 was selected to approximate scale‐free topology (Figure S1A), resulting in 34 gene modules (Figure S1B). Correlating module eigengenes with CytoTRACE scores identified the turquoise module as most strongly associated with stemness (Figure 2C). Overlap between genes in this module and malignant‐cell markers (average log fold change > 0.5, *p* < 0.05) produced a set of 127 tumor‐enriched genes, which we defined as the CSC signature (Figure 2D; Table S1). This signature included several well‐established stemness‐associated genes, such as EPCAM and EGFR. CSC activity quantified using AUCell showed consistent agreement with CytoTRACE scores (correlation ∼0.8) and was reproducible across three independent NSCLC datasets (GSE127465, GSE143423, and EMTAB6149) (Figure 2E).

### 2.2 DSG2 Had Negative Roles in Chemotherapy and EGFR‐TKI Therapy

**FIGURE 3:**
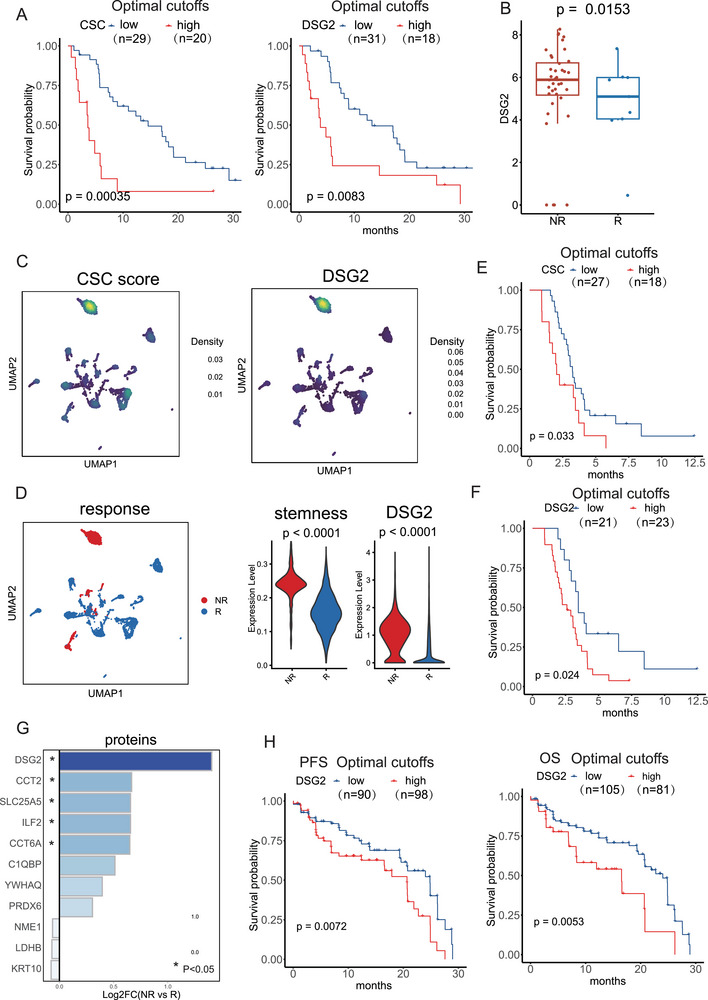
The adverse role of cancer stem cells (CSC) in chemotherapy and EGFR‐TKI therapy. (A) NSCLC patients in this cohort with higher CSC score or DSG2 expression exhibited shorter OS. (B) DSG2 displayed higher expression in both non‐responders (NR) and responders (R). (C) Distribution of CSC score and DSG2 in patients treated with EGFR‐TKI therapy in single‐cell level. (D) Both the CSC score and DGS2 had elevated expression in NR patients (E) The CSC score showed significant adverse association with worse clinical outcome in EGFR‐TKI‐treated patients. (F) DGS2 exhibited significant adverse association with worse clinical outcome in EGFR‐TKI‐treated patients. (G) Among all 521 proteins in the proteomic data, 11 proteins were present in the CSC signature, and their expression in NR and R is shown. (H) Serum DSG2 demonstrated an adverse association with a worse clinical outcome in patients treated with EGFR‐TKI therapy in the BPI‐7711 clinical trial. Patients were divided into high and low expression groups using an optimal cut‐off determined by maximally selected rank statistics. The number of patients in each group is indicated.

Consequently, we examined the role of CSCs in predicting the treatment effectiveness for patients receiving anti‐tumor therapies. We integrated large‐scale transcriptomics data from the ORIENT‐3 clinical trial (NCT03150875) [28]. The dataset included 49 patients with NSCLC who received chemotherapy and 61 NSCLC patients who received immunotherapy. Upon initial examination of the chemotherapy subset, we discovered that NSCLC patients with higher CSC scores or greater levels of DSG2 had shorter OS times (Figure 3A). DSG2, a CSC marker, exhibited elevated expression levels in both non‐responders (NR) and responders (R) (Figure 3B). Survival analysis demonstrated that higher DSG2 expression was significantly associated with worse clinical outcomes, with a hazard ratio (HR) of 1.35 (p = 0.0044). Receiver operating characteristic (ROC) analysis further supported the predictive value of DSG2 for chemotherapy response, yielding an area under the curve (AUC) of 0.812 (p = 0.015).

We then conducted a thorough analysis using multi‐omics data to investigate the clinical implications of CSCs in NSCLC patients who received EGFR‐TKI therapy. Initially, we collected single‐cell data from 49 clinical biopsies of 30 patients with metastatic lung cancer before and during targeted therapy [29]. Using the AUCell R program, we computed the CSC score in each tumor cell and observed that the expression pattern of DSG2 closely resembled the CSC score, suggesting that DSG2 accurately captures stemness characteristics (Figure 3C). Both the CSC score and DSG2 exhibited increased expression in NR patients (Figure 3D). Additionally, we acquired mRNA data from a group of 44 patients who received targeted therapy as part of the IMPACT research (https://src.gisapps.org/OncoSG/). The CSC score and DSG2 marker showed a noteworthy negative correlation with poorer clinical outcomes in individuals treated with EGFR‐TKIs (Figure 3E,F).

We further examined serum proteomic and clinical data from a prospective cohort. This group consisted of 186 patients with locally advanced or metastatic NSCLC in BPI‐7711 clinical trail (NCT03386955) [28]. Quantification of 521 proteins in patient plasma samples obtained at baseline was performed using mass spectrometry. Among the 186 patients, 57 (30.65%) did not experience clinical improvement, while 129 (69.35%) derived benefits from the medication. Out of the 521 proteins in the proteomic data, 11 proteins were present in CSC signature. DSG2 showed the most significant increase in expression of the 11 proteins in NR compared to R (Figure 3G). DSG2 also had a notable negative correlation with poorer clinical outcomes in patients treated with EGFR‐TKI (Figure 3H). Elevated circulating DSG2 levels were strongly associated with inferior therapeutic outcomes, with an HR of 2.17 (p = 0.00014). ROC analysis demonstrated robust discriminatory performance, with an AUC of 0.913 (p = 0.00047). These findings suggest that DSG2 has potential as a biomarker for predicting treatment outcomes.

**FIGURE 4:**
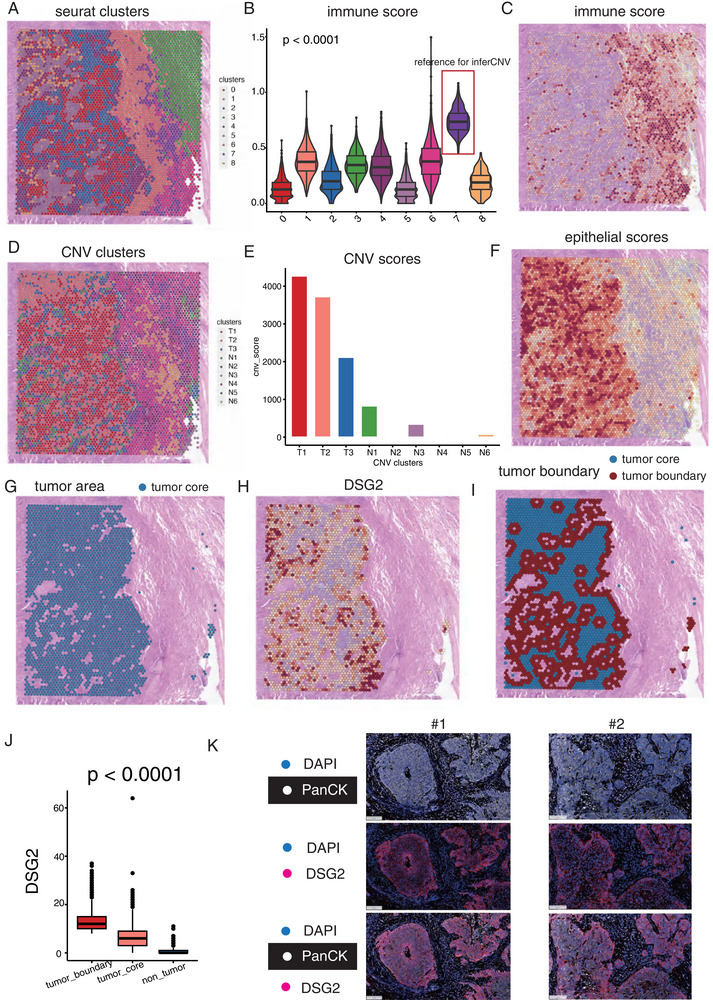
Cancer stem cells (CSC) mainly located at the tumor boundary revealed by spatial transcriptomics. (A) Clustering of 4931 spots in sample 1 into 8 distinct clusters. (B) Distribution of immune score in the 8 clusters. (C) The feature plot displayed the distribution of immune score in sample 1. (D) Hierarchical clustering assigning all spots in sample 1, except the reference cluster, into eight clusters. (E) Bar charts showing the distribution of copy number variation (CNV) score in the nine clusters. (F) The feature plot displayed the distribution of epithelial score in sample 1. (G) The plot depicted the tumor area. (H) Plot showing the distribution of DSG2 sample 1. (I) The plot depicted the tumor core and the tumor boundary, defined as the nearest 2‐spot width area near the outermost circle of the tumor area. (J) The expression of DSG2 in the tumor core and tumor boundary area. (K) The multiplex immunofluorescence image of DSG2 in tumor samples.

### 2.3 DSG2+ CSC Co‐Located With FAP+ myCAF in the Tumor Boundary

To examine the spatial distribution of CSCs, spatial transcriptomic sequencing was conducted on tumor tissue slices obtained from 6 patients with NSCLC. The quality data for these samples is detailed in Table S2. Distinguishing malignant cells solely based on gene expression patterns in ST data poses challenges, particularly in distinguishing them from normal epithelial cells. To address this complexity, we employed inferCNV analysis to accurately differentiate cancerous cells by analyzing their copy number variation patterns.

The process began with identifying reference cells for the inferCNV pipeline. These reference cells were selected based on their immune scores, calculated using immune‐related signatures including pan‐immune markers (PTPRC), T cell markers (CD2, CD3D, CD3E, CD3G), B cell markers (CD79A, MS4A1, CD79B), and myeloid cell markers (CD68, CD14). Cluster 7, identified with the highest immune score among 4931 spots in sample 1, was chosen as the reference cluster (Figure 4A and 4B). Figure 4C illustrates the distribution of immune scores across the sample. In the second clustering phase, hierarchical clustering was used to analyze CNV patterns and distinguish malignant cells from other cell types. Clusters 1, 2, and 3, characterized by significantly elevated CNV scores, were identified as malignant clusters (Figure 4D and 4E). Validation of these annotations was performed through histological examination by pathologists and analysis of tumor‐related epithelial markers (EPCAM, KRT8, KRT19), confirming accurate identification of tumor regions (Figure 4F).

Subsequent analysis focused on the expression pattern of DSG2, which showed predominant expression in the tumor areas (Figure 4G and 4H). Notably, DSG2 expression was highest at the tumor boundary, indicating its association with invasive characteristics of DSG2+ CSCs (Figure 4I and 4J). Further validation using multiplex immunofluorescence analysis on 20 NSCLC tumor samples confirmed elevated DSG2 expression at the tumor boundary compared to the core, highlighting its relevance in identifying CSCs with invasive potential (Figure 4K).

**FIGURE 5:**
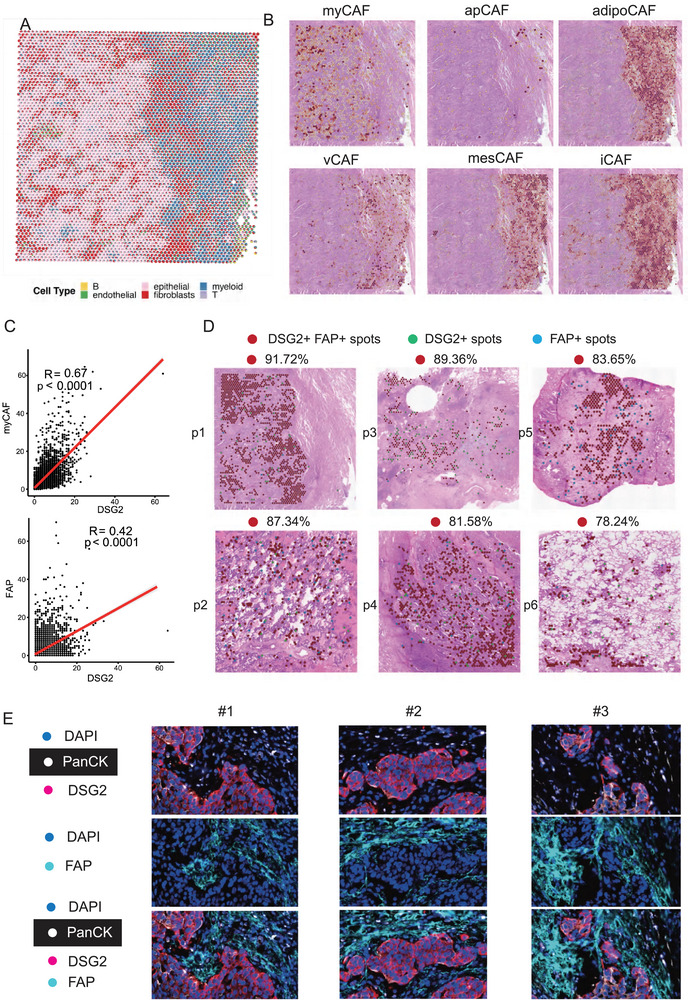
Co‐location of DSG2+ CSC and FAP+ myCAF revealed by spatial transcriptomics. (A) The cell type deconvolution of six cell types (epithelial cells, myeloid cells, fibroblasts, endothelial cells, T cells, and B cells) in each spot. (B) The infiltration of six subtypes of tumor‐associated fibroblasts (CAF): myCAF, inflammatory CAFs (iCAFs), antigen‐presenting CAFs (apCAFs), vascular CAFs (vCAFs), mesothelial CAF (mesCAF), and adipogenic CAF (adipoCAF). (C) The co‐localization between DSG2+ CSC and FAP+ myCAF in the five samples (D) The correlation between DSG2+ CSC and FAP+ myCAF in bulk level. (E) The multiplex immunofluorescence image of FAP and DSG2 in tumor samples.

To elucidate the spatial organization associated with CSCs, we utilized cell type deconvolution through the CARD R package [30]. This approach integrates cell‐type‐specific expression data from single‐cell RNA sequencing to infer cellular composition across tissue regions. Initially, we categorized spots into six primary cell types—epithelial cells, myeloid cells, fibroblasts, endothelial cells, T cells, and B cells (Figure 5A). These results were consistent with HE pathological annotations, revealing fibroblasts as the predominant cell type infiltrating the tumor area, followed by myeloid cells and endothelial cells. Immune cells such as B cells and T cells were sparsely distributed within the tumor, with fibroblasts prominently surrounding the tumor, potentially supporting the maintenance of CSCs and suggesting a pro‐tumor role.

Given the diverse functions of cancer‐associated fibroblasts (CAFs) in the TME, we investigated specific subtypes of CAFs present in the tumor region. Selected markers for six CAF subtypes—myofibroblasts (myCAF), inflammatory CAFs (iCAFs), antigen‐presenting CAFs (apCAFs), vascular CAFs (vCAFs), mesothelial CAFs (mesCAF), and adipogenic CAFs (adipoCAF)—were analyzed (Table S3). Our findings indicated that myCAF subtype showed exclusive infiltration within the tumor area, while the other subtypes were more abundant in neighboring normal tissue (Figure 5B).

We further explored the correlation between DSG2 expression and the myCAF signature, revealing a high correlation coefficient (R = 0.67, *p*<0.001) (Figure 5C). Among the typical markers of myCAF, FAP exhibited the strongest correlation with DSG2 (R = 0.42, *p*<0.001) (Figure 5C). We labeled the gene expression of DSG2 and FAP greater than zero as DSG2+FAP+ spots, indicating the co‐location of DSG2+ tumor cells and FAP+ CAF. Additionally, spots with either DSG2 or FAP expression greater than zero were labeled as DSG2+ spots or FAP+ spots, respectively. The distribution of these three types of spots in Figure 5D shows that DSG2+FAP+ spots are the majority among the spots expressing DSG2 and FAP. The DSG2+ spots and FAP+ spots are situated around the DSG2+FAP+ spots, highlighting a strong co‐localization pattern of DSG2+ tumor cells and FAP+ CAF (Figure 5D). Across all samples, DSG2^+^FAP+spots accounted for 78.24%–91.72% of the DSG2‐ or FAP‐expressing spots, indicating that the majority of DSG2‐ and FAP‐positive spots exhibit concurrent expression of both genes. Continual observation across samples confirmed co‐localization of DSG2+ CSCs and FAP+ myCAFs, suggesting physical interaction within the niche (Figure 5D). To validate these findings at the protein level, multiplex immunofluorescence analysis was performed on 20 NSCLC tumor samples. Using panCK to identify tumor cells, DSG2 to identify CSCs, and FAP to identify myCAFs, we confirmed a robust spatial relationship between DSG2+ CSCs and FAP+myCAFs at the tumor boundary, implying potential communication between these cell types (Figure 5E). These results underscore the intricate interplay between CSCs and specific CAF subtypes, particularly myCAFs, in shaping the tumor microenvironment and influencing disease progression.

### 2.4 DSG2+ CSC and FAP+ myCAF Had Negative Roles in Immunotherapy

**FIGURE 6:**
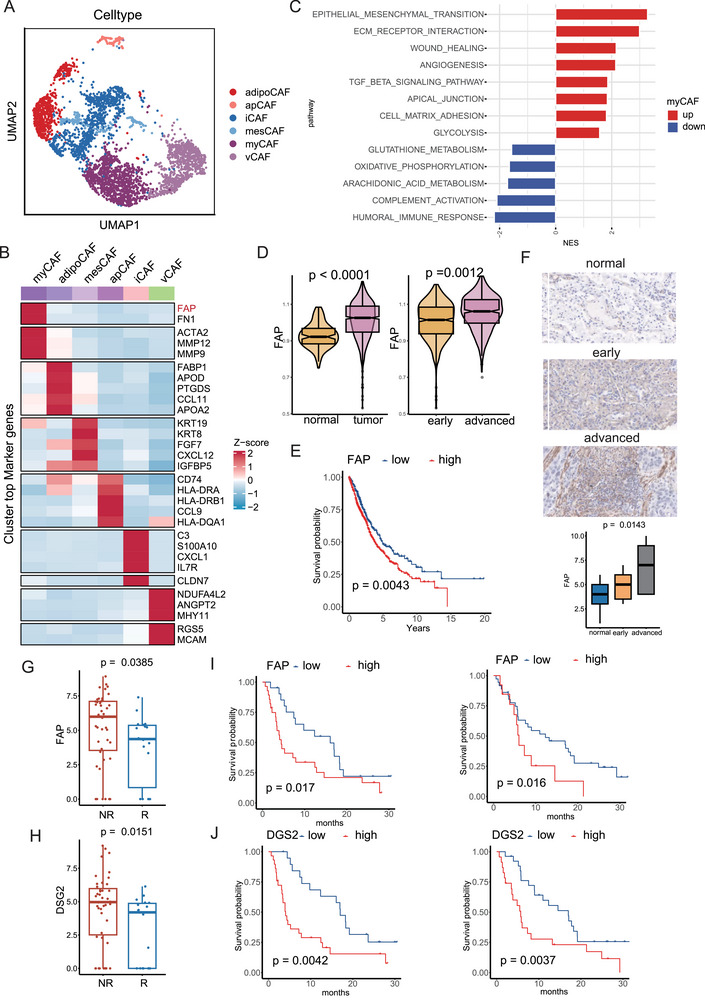
DSG2+ CSC and FAP+ myCAF had negative roles in immunotherapy. (A) uniform manifold approximation and projection (UMAP) plots showing six subtype of tumor‐associated fibroblasts (CAF), including myCAF, iCAF, apCAF, adipoCAF, vCAF and mesCAF in non‐small cell lung cancer (NSCLC). (B) The differential expressed genes (DEGs) of six subtype of CAF (C) Bar chart displaying the up‐regulated and down‐regulated pathways in FAP+ myCAF. (D) Distribution of FAP+ myCAF in normal and tumor tissues (left) and distribution of FAP+ myCAF in early and advanced stage NSCLC tissues. (E) NSCLC patients with higher expression of FAP exhibited shorter overall survival (OS). (F) Immunohistochemistry images of FAP in our cohort consisting of 70 NSCLC patients. (G) The non‐responders of exhibited higher FAP compared to responders in NSCLC patients treated with immunotherapy. (H) The non‐responders of exhibited higher DSG2 compared to responders in NSCLC patients treated with immunotherapy. (I) Patients with higher FAP had significantly worse therapeutic results. (J) Patients with higher DSG2 had significantly worse therapeutic results.

To precisely delineate the characteristics of FAP+ myCAF, we conducted a comprehensive study examining different CAFs at the single‐cell level using the GSE148071 dataset. Initially, we classified fibroblasts (n = 3928) into six distinct subclusters: myCAF, iCAF, apCAF, adipoCAF, vCAF, and mesCAF (Figure 6A). Notably, myCAF emerged with unique functional attributes compared to other fibroblast subtypes, characterized by high expression of FAP. Moreover, myCAF exhibited elevated levels of metalloproteinases such as MMP9 and MMP12, crucial for extracellular matrix remodeling and immune response modulation (Figure 6B). Differential gene expression analysis revealed that myCAF was enriched in pathways related to EMT, angiogenesis, TGFB signaling, and glycolysis, while immune‐related pathways like complement activation and humoral immune response were notably suppressed in myCAF (Figure 6C). These findings suggest that myCAF may play a role in promoting EMT and suppressing immune responses in NSCLC.

Examining data from the TCGA cohort further underscored the clinical relevance of FAP+ myCAF. We observed a significant increase in FAP+ myCAF infiltration in tumor samples compared to adjacent healthy tissues, with higher levels correlating with advanced stages of NSCLC and poorer OS outcomes in NSCLC patients (Figure 6D–F). Given the pivotal roles of CSCs and myCAF in influencing treatment responses, we investigated the predictive potential of DSG2+ CSC and FAP+ myCAF in patients undergoing immunotherapy using data from the ORIENT‐3 clinical trail [31]. Analysis revealed that individuals with higher levels of CSCs and myCAF were associated with poorer responses to immunotherapy (Figure 6G–J). These findings underscore the importance of CSCs and myCAF as potential biomarkers for predicting immunotherapy outcomes in NSCLC patients, highlighting their relevance in clinical decision‐making.

### 2.5 Cell‐Cell Communication Between DSG2+ CSC and FAP+ myCAF

**FIGURE 7:**
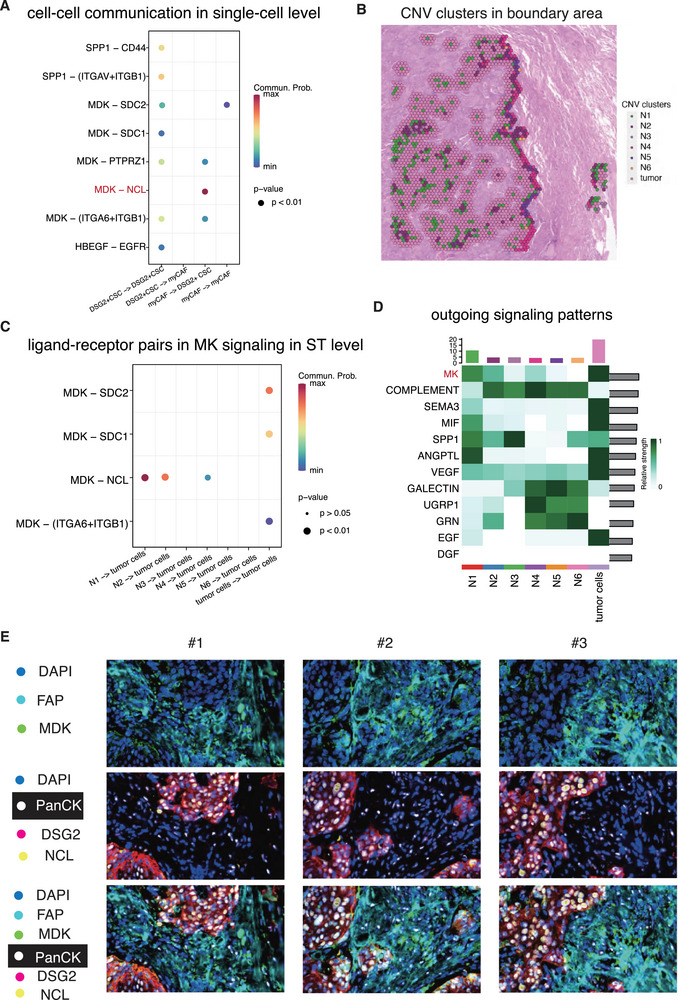
Cell‐cell communication between DSG2+ CSC and FAP+ myCAF. (A) The cell‐cell communication between DSG2+ CSC and FAP+ myCAF at the single‐cell level. (B) The CNV clusters in the boundary area. (C) The ligand‐receptor pairs in MK signaling pathway signaled from all clusters to tumor cells. (D) The outgoing pattern of tumor cells and other non‐tumor clusters in the boundary area. (E) The multiplex immunofluorescence image revealed the MDK‐NCL ligand‐receptor pair in the cell‐cell communication DSG2+ CSC and FAP+ myCAF.

In our study, we investigated the interactions between DSG2+ CSCs and FAP+ myCAFs, focusing on their co‐location at the boundary area of the tumor. Recognizing precise interactions between DSG2+ CSCs and FAP+ myCAFs was challenging due to the limitation of the 10X Genomics Visium platform, which accommodates 1–10 cells per spot. Therefore, we initially evaluated their putative crosstalk at the single‐cell level. Our analysis identified the MK and SPP1 signaling pathways as key mediators of communication between these cell types (Figure 7A). In the self‐communication of DSG2+ CSCs, SPP1 interacted with CD44, ITGAV, and ITGB1. Midkine (MDK), a key ligand of the MK signaling pathway, has been implicated in supporting cancer progression in various contexts. Our findings indicated that MDK‐SDC1, MDK‐SDC2, MDK‐PTPRZ1, and MDK‐(ITGA6+ITGB1) actively participated in the self‐communication of DSG2+ CSCs. Furthermore, MDK released from FAP+ myCAFs activated nucleolin (NCL) in DSG2+ CSCs. NCL, a major nucleolar protein of growing cells, also functions as a cell surface receptor, shuttling between the cytoplasm and nucleus, thereby providing a mechanism for extracellular regulation of nuclear events.

Given the co‐location of DSG2+ CSCs with FAP+ myCAFs at the tumor boundary, we concentrated our analysis on intercellular communication in this area. The boundary area was defined as the nearest 2‐spot width region near the outermost circle of the tumor boundary line, encompassing all clusters (Figure 7B). In this region, MDK was found to activate NCL in DSG2+ CSCs, with FAP+ myCAFs releasing MDK in the N1 and N2 clusters. The MK signaling pathway exhibited the highest relative strength among all communication activities (Figure 7D). To validate our findings at the protein level, we performed multiplex immunofluorescence on 20 tumor samples from NSCLC patients, using panCK antibody to annotate tumor cells, DSG2 to annotate CSCs, and FAP to annotate myCAFs. The results demonstrated that MDK, a secretory protein from FAP+ myCAFs, activated NCL in DSG2+ CSCs, thereby confirming the specific cell‐cell interaction of this ligand‐receptor pair (Figure 7E). These findings consistently supported our single‐cell and spatial transcriptomics results, emphasizing the critical role of MDK's interaction with NCL on DSG2+ CSCs in the tumor boundary area.

### 2.6 DSG2 Promotes CSC‐Associated Functional Properties and Proliferative Capacity in NSCLC Cells

**FIGURE 8:**
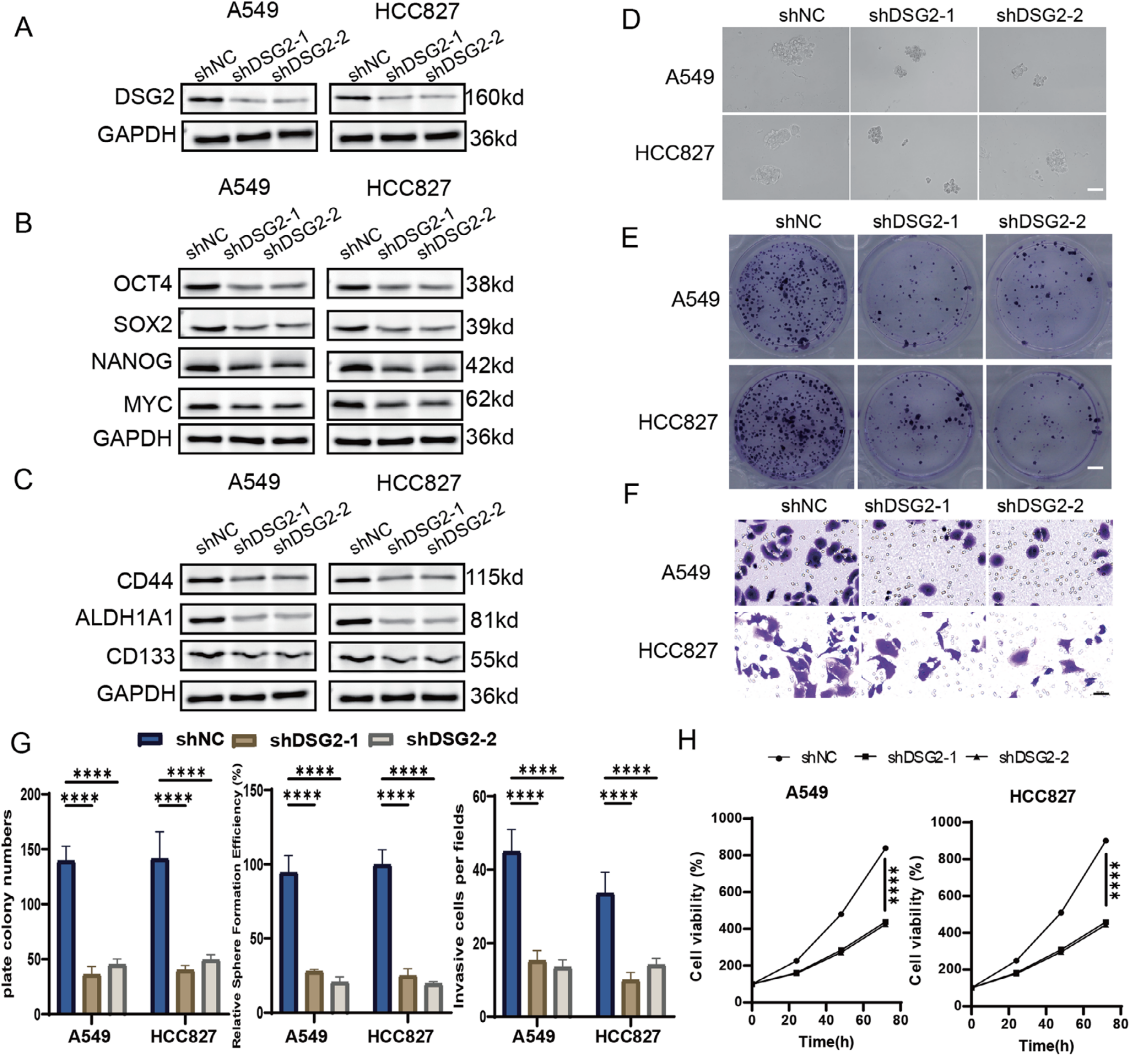
DSG2 is functionally required for maintaining CSC‐associated properties and proliferative capacity in NSCLC cells. (A) Immunoblot analysis confirming efficient DSG2 knockdown in A549 and HCC827 cells using two independent shRNAs (shDSG2‐1 and shDSG2‐2). GAPDH was used as a loading control. (B) Western blot analysis of stemness‐associated transcription factors (OCT4, SOX2, NANOG, and MYC) in control and DSG2‐silenced A549 and HCC827 cells. (C) Western blot analysis of canonical CSC markers (CD44, ALDH1A1, and CD133) following DSG2 knockdown in A549 and HCC827 cells. (D) Representative images of tumorsphere formation assays showing reduced sphere size and number in DSG2‐silenced cells compared with control cells. Scale bar, 500 µm. (E) Representative images of single‐cell clonogenic assays demonstrating decreased colony formation upon DSG2 depletion. Scale bar, 500 µm. (F) Representative images of Transwell invasion assays showing reduced invasive capacity in DSG2 knockdown cells. Scale bar, 500 µm. (G) Quantification of plate colony numbers, relative sphere‐forming efficiency, and invasive cell numbers per field in A549 and HCC827 cells. Data are presented as mean ± SD. (H) Cell viability curves assessed by CCK‐8 assays showing reduced proliferative capacity in DSG2‐silenced cells compared with controls over time. ^*^Statistical significance: ^*^ *p* < 0.05, ^**^ *p* < 0.01, ^***^ *p* < 0.001, ^****^ *p* < 0.0001.

To define the functional role of DSG2 in NSCLC, DSG2 was silenced in A549 and HCC827 cells using two independent shRNAs. Immunoblotting confirmed efficient DSG2 knockdown (Figure 8A), which was accompanied by marked downregulation of stemness‐associated transcription factors (OCT4, SOX2, NANOG, and MYC; Figure 8B) and canonical CSC markers (CD44, ALDH1A1, and CD133; Figure 8C).

Functionally, DSG2 depletion significantly impaired CSC‐associated properties. Tumorsphere formation assays showed a pronounced reduction in sphere size and number in DSG2‐silenced cells (Figure 8D), and single‐cell clonogenic assays revealed markedly decreased colony‐forming capacity (Figure 8E). Quantitative analyses confirmed significant reductions in plate colony numbers and relative sphere‐forming efficiency in both cell lines (Figure 8G). In addition, DSG2 knockdown substantially suppressed invasive potential, as demonstrated by Transwell invasion assays and corresponding quantification (Figure 8F,G), indicating a role for DSG2 in promoting invasive CSC‐associated behaviors. Cell viability assays further revealed that DSG2 depletion also reduced proliferative capacity over time (Figure 8H), suggesting that DSG2 contributes to tumor cell growth in addition to regulating CSC‐associated functional properties.

Together, these data demonstrate that DSG2 is functionally required for maintaining CSC‐associated molecular programs, self‐renewal capacity, invasiveness, and proliferative potential in NSCLC cells.

### 2.7 FAP+myCAFs Functionally Enhance CSC‐Associated Properties of DSG2^High Tumor Cells Through MMP‐Dependent Paracrine Signaling

**FIGURE 9:**
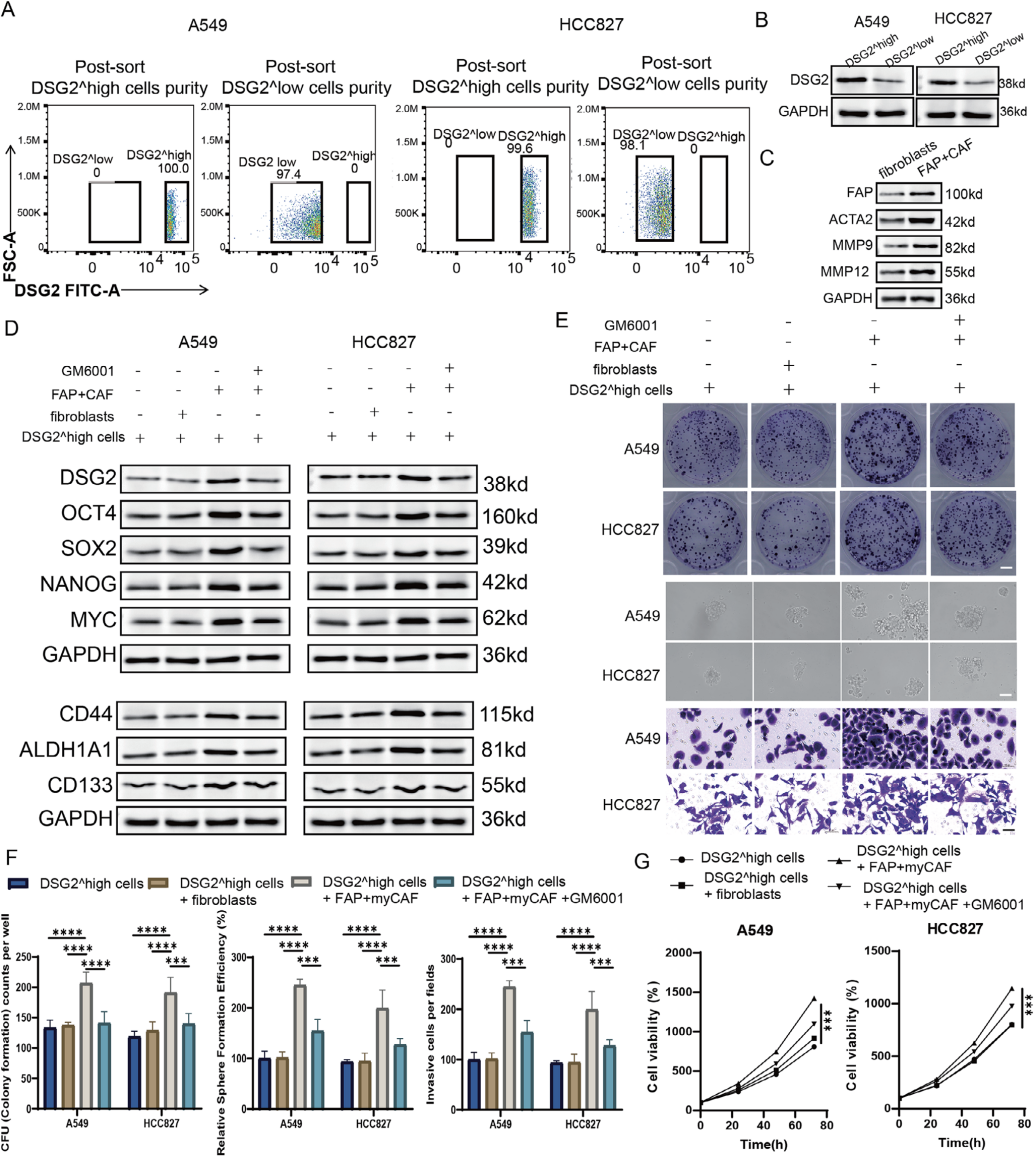
FAP+myCAFs enhance CSC‐associated properties of DSG2^high tumor cells through MMP‐dependent paracrine signaling. (A) FACS gating strategy and post‐sort purity analysis for isolation of DSG2^high and DSG2^low tumor cell populations from A549 and HCC827 cells. The top and bottom 10% of DSG2‐expressing cells were collected as DSG2^high and DSG2^low fractions, respectively. (B) Western blot validation of DSG2 protein levels in sorted DSG2^high and DSG2^low populations. GAPDH was used as a loading control. (C) Western blot confirmation of fibroblast differentiation into FAP+myCAFs, showing increased expression of FAP, ACTA2, MMP9, and MMP12. GAPDH was used as a loading control. (D) Western blot analysis of stemness‐associated transcription factors (OCT4, SOX2, NANOG, and MYC) and CSC markers (CD44, ALDH1A1, and CD133) in DSG2^high tumor cells under Transwell co‐culture conditions: tumor cells alone, co‐culture with control fibroblasts, co‐culture with FAP+myCAFs, and co‐culture with FAP+myCAFs plus GM6001, as indicated. (E) Representative images of clonogenic assays, tumorsphere formation assays, and Transwell invasion assays for DSG2^high tumor cells under the indicated co‐culture conditions. Scale bar, 500 µm. (F) Quantification of colony formation units (CFU), relative sphere‐forming efficiency, and invasive cell numbers per field in A549 and HCC827 DSG2^high tumor cells across the indicated conditions. Data are presented as mean ± SD. (G) Cell viability curves assessed by CCK‐8 assays showing the effects of control fibroblasts, FAP+myCAFs, and GM6001 on the proliferative capacity of DSG2^high tumor cells over time. ^*^Statistical significance: ^*^ *p* < 0.05, ^**^ *p* < 0.01, ^***^ *p* < 0.001, ^****^ *p* < 0.0001.

To functionally test CSC–fibroblast interactions, DSG2^high and DSG2^low tumor cell populations were isolated from A549 and HCC827 cells by fluorescence‐activated cell sorting (FACS). Cells were ranked by DSG2 fluorescence intensity, and the top and bottom 10% fractions were collected as DSG2^high and DSG2^low populations, respectively. Post‐sort analysis confirmed high purity of the isolated subsets in both cell lines (Figure 9A), and western blotting further validated markedly higher DSG2 protein abundance in DSG2^high cells compared with DSG2^low cells (Figure 9B).

To establish a stromal model, fibroblasts were differentiated into FAP+myCAFs, as confirmed by increased expression of FAP, ACTA2 (α‐SMA), MMP9, and MMP12 (Figure 9C). DSG2^high tumor cells were then subjected to Transwell co‐culture with either control fibroblasts or FAP+myCAFs, with or without the broad‐spectrum MMP inhibitor GM6001. Co‐culture with FAP+myCAFs substantially increased the expression of stemness‐associated transcription factors (OCT4, SOX2, NANOG, and MYC) and canonical CSC markers (CD44, ALDH1A1, and CD133) in DSG2^high tumor cells, whereas control fibroblasts exerted minimal effects (Figure 9D). Importantly, GM6001 markedly attenuated the myCAF‐induced upregulation of these CSC‐associated markers, indicating partial dependence on MMP activity.

Consistent with these molecular changes, FAP+myCAF co‐culture significantly enhanced clonogenic growth, tumorsphere formation, and invasive capacity of DSG2^high tumor cells in both A549 and HCC827 cells, and these effects were reduced by GM6001 treatment (Figure 9E,F). In parallel, CCK‐8 assays showed that FAP+myCAFs also promoted the proliferative capacity of DSG2^high tumor cells, which was partially diminished upon MMP inhibition (Figure 9G). Together, these data demonstrate that FAP+myCAFs actively promote CSC‐associated phenotypes and tumor cell growth in DSG2^high tumor cells through MMP‐dependent paracrine mechanisms, supporting a functional CSC–myCAF interaction beyond spatial co‐localization.

## 3 Discussion

CSCs drive tumor progression, metastasis, and therapeutic resistance largely by shaping an immunosuppressive TME [32]. However, their spatial organization and functional interactions in NSCLC remain insufficiently defined. Here, we established a single‐cell‐derived CSC gene signature and examined their spatial association with myCAFs. We further evaluated the relationship between CSC–myCAF niches and immunotherapy outcomes, highlighting their potential value as predictive biomarkers and therapeutic targets.

DSG2, a constituent of desmosomal cell‐cell adhesion structures found in epithelial tissues, plays pivotal roles in various biological processes, including EMT, cell proliferation, and migration [33, 34]. Recent research has highlighted its involvement in enhancing the malignant properties of stem cells by activating the Wnt/β‐catenin signaling pathway. In cancers like cutaneous squamous cell carcinoma, DSG2 contributes to the formation of extracellular vesicles, impacting tumor invasion and drug resistance, and serving as a prognostic and therapeutic predictor [35]. Additionally, DSG2 promotes tumor growth, facilitates clustering of circulating tumor cells, and supports metastasis to distant organs [36]. Its interaction with hypoxia influences cancer cell dissemination in breast cancer, correlating with poor prognosis and increased recurrence risk in patients [37].

Consistent with the dynamic CSC model, DSG2 should not be viewed as an exclusive or static CSC marker. Instead, its expression likely reflects a plastic stem‐like state that tumor cells can acquire or lose in response to microenvironmental cues. This interpretation is further supported by spatial transcriptomics and multiplex immunofluorescence analyses, which revealed that DSG2+ CSCs are enriched at the tumor boundary and spatially co‐localized with FAP+ myofibroblasts, a niche implicated in CSC maintenance. Moreover, immunohistochemical validation across an independent cohort demonstrated that DSG2 expression is significantly elevated in tumor epithelium compared with adjacent normal tissue, reinforcing its tumor‐biased and CSC‐associated nature.

Despite this, developing DSG2‐targeted strategies may still be feasible for several reasons. First, many tumors show markedly elevated and/or more homogeneous DSG2 expression compared with most normal tissues, which may provide a therapeutic window, particularly in molecularly selected patients with high DSG2 expression. Second, multiple modality and engineering approaches could mitigate normal‐tissue liability, including i) antibody–drug conjugates (ADCs) or radioligand approaches that rely on differential antigen density/internalization to preferentially affect DSG2‐high tumor cells, ii) affinity‐tuned antibodies/CARs or logic‐gated designs to reduce recognition of low‐level DSG2 in normal tissues, and iii) tumor‐restricted delivery strategies (e.g., local delivery or conditionally activated/prodrug‐like formats) to further improve safety.

In the context of TME modulation by CSCs, our investigation has revealed insights into their role in tumor immunity. CAFs, prominent stromal cells in the TME, are critical in conferring resistance to therapies through diverse mechanisms [38, 39]. Specifically, CAFs in NSCLC sustain CSC stemness via paracrine signaling pathways, particularly highlighted in co‐culture experiments [40]. Notably, myCAFs, found in close proximity to cancer cells, suggest potential juxtacrine interactions that maintain an immunosuppressive and tumor‐supportive microenvironment [41]. These insights underscore the complex interplay between DSG2, CSCs, and CAFs in shaping the TME and influencing cancer progression and therapeutic responses. Understanding these interactions holds promise for developing targeted therapies aimed at disrupting these pathways to mitigate tumor aggressiveness and improve patient outcomes.

Our study suggests several avenues for therapeutic intervention targeting the interactions between DSG2+ CSCs and FAP+ myCAF as well as disrupting the MDK‐NCL signaling pathway. Given the role of DSG2 in therapy resistance and as a serum marker for therapy response, monoclonal antibodies targeting DSG2 could be developed to selectively eliminate DSG2+ CSCs, potentially enhancing the effectiveness of existing chemotherapies and targeted therapies. Additionally, small molecule inhibitors designed to disrupt DSG2 function could prevent the maintenance and survival of CSCs, thereby reducing tumor aggressiveness and resistance to treatment. Combining therapies that target DSG2+ CSCs with those that inhibit FAP+ myCAF could disrupt the supportive microenvironment that sustains CSCs. For instance, antifibrotic agents targeting FAP could be used alongside DSG2‐targeted therapies to dismantle the protective niche for CSCs. Therapeutic agents that inhibit the physical interaction between DSG2+ CSCs and FAP+ myCAF could also be developed, which might include molecules that interfere with adhesion pathways or disrupt signaling molecules involved in their interaction. As our spatial analysis identified MMP9 and MMP12 expression by FAP+ myCAFs, using MMP inhibitors could prevent the EMT and invasive behavior of CSCs, reducing tumor invasiveness and improving response to therapies.

Our study identified the MDK‐NCL signaling pathway as highly active in cell‐cell communication at the tumor boundary. Inhibitors targeting MDK or its receptor NCL could be developed to disrupt this pathway, potentially impairing the supportive signaling network for CSCs and enhancing the effectiveness of immunotherapies. Strategies to reduce or block the production of MDK could diminish its signaling activity and impact on CSC maintenance. We propose conducting preclinical studies to evaluate the efficacy of the aforementioned therapeutic strategies in vitro and in vivo, determining the potential of targeting DSG2+ CSCs, FAP+ myCAF, and the MDK‐NCL signaling pathway in reducing tumor growth and improving treatment responses. Based on promising preclinical results, early‐phase clinical trials could be designed to test the safety and efficacy of these novel therapeutic approaches in patients with cancer types exhibiting high levels of DSG2+ CSCs and FAP+ myCAF.

Several limitations should be considered. Associations between DSG2 expression and treatment response were derived from retrospective analyses of clinical cohorts and require independent and prospective validation. The functional consequences of CSC–myCAF co‐localization were inferred from spatial and transcriptomic data and remain to be experimentally confirmed. Moreover, additional orthogonal approaches, such as flow cytometry or independent single‐cell platforms, would strengthen validation. Despite these limitations, our study provides a spatial and functional framework for understanding CSC‐driven resistance in NSCLC and identifies actionable targets for therapeutic intervention.

## 4 Materials and Methods

### 4.1 Patient Samples

Formalin‐fixed paraffin‐embedded (FFPE) specimens were collected from 70 untreated non‐small cell lung cancer (NSCLC) patients at the Cancer Hospital, Chinese Academy of Medical Sciences (Beijing, China). All samples were obtained with informed consent and in accordance with institutional ethical guidelines. The study protocol was approved by the Ethics Committee of Institut Curie (Approval No. 23/262‐4004). Among these samples, all 70 were used for immunohistochemistry, and six representative cases were further subjected to spatial transcriptomics analysis.

### 4.2 Data Sources

Multiple publicly available datasets were integrated to characterize NSCLC at both single‐cell and bulk levels. Single‐cell RNA sequencing data from GSE148071 (172,164 cells from 57 tumors) were used as the primary dataset, with GSE127465, GSE143423, and EMTAB6149 serving as validation cohorts. Additional single‐cell data from 49 samples of 30 patients receiving EGFR‐TKI therapy were included to assess treatment‐associated changes. Bulk transcriptomic and clinical data were obtained from The Cancer Genome Atlas (TCGA), while mRNA expression profiles from 44 targeted‐therapy‐treated patients were retrieved from the IMPACT project.

### 4.3 Dimension Reduction and Cell Clustering

Highly variable genes (top 2000) were identified using the FindVariableFeatures function and subjected to principal component analysis. Cell clustering was performed using the FindNeighbors and FindClusters functions. Major cell types were annotated based on canonical markers, including epithelial (EPCAM, KRT8, KRT19), fibroblast (COL1A1, COL1A2, DCN), endothelial (PLVAP, VWF, PECAM1), T cell (CD3D, CD3E, TRAC), B cell (MS4A1, CD79A), and myeloid (CD14, CD163, CD68, FCGR3A) markers.

### 4.4 InferCNV Analysis

Large‐scale chromosomal copy number variations were inferred using InferCNV. Fibroblasts and endothelial cells were used as reference populations. InferCNV objects were generated from raw count matrices, cell annotations, and gene position files, and analyses were conducted with default settings (cutoff = 0.1, cluster_by_groups = TRUE, denoise = TRUE, HMM = FALSE).

### 4.5 CytoTRACE Analysis

Cell differentiation status was estimated using the CytoTRACE algorithm, which assigns each cell a score reflecting transcriptional diversity and stemness. CytoTRACE scores for malignant cells were calculated with the CytoTRACE R package, ranging from 0 to 1, with higher values indicating lower differentiation and increased stem‐like features.

### 4.6 Definition of Cancer Stemness

Given the plastic and reversible nature of cancer stemness, cancer stem cells were not defined as a discrete population. Instead, stemness was treated as a continuous trait across malignant cells, quantified by CytoTRACE scores, where higher scores denote stronger stem‐like potential.

### 4.7 Transcription Factor Activity Inference

Transcription factor (TF) activity was inferred using the DoRothEA framework based on curated TF–target interactions. Human DoRothEA regulons with confidence levels A–C were selected, and regulon activity was estimated using the VIPER algorithm via the run_viper function, integrating expression levels of TFs and their targets.

### 4.8 Metacell Analysis

Tumor cells were aggregated into metacells using the R metacell package. Mitochondrial genes were excluded prior to analysis. Genes with scaled variance greater than 0.08 were selected to compute cell–cell similarity. K‐nearest neighbor graphs (K = 100) were constructed, followed by 500 resampling iterations using 75% of cells to generate coclustering graphs with a minimum metacell size of 50. Metacell‐level gene expression and CytoTRACE scores were calculated by averaging values from constituent cells.

### 4.9 WGCNA

The R package WGCNA was applied to identify gene modules associated with cancer stemness. The pickSoftThreshold function was used to select the optimal soft‐thresholding power for network construction. A weighted adjacency matrix was generated, followed by hierarchical clustering based on the topological overlap matrix (1 − TOM) to define gene modules. Module–trait correlations were then calculated against CytoTRACE scores.

### 4.10 AUCell

Gene expression rankings for each cell were first generated using AUCell_buildRankings with default settings. The AUCell_calcAUC function subsequently computed area‐under‐the‐curve (AUC) scores to quantify enrichment of cancer stem cell (CSC)‐related gene signatures. Higher AUC values indicated stronger CSC‐like transcriptional programs.

### 4.11 Plasma Proteomics in Patients Receiving Third‐Generation EGFR‐TKI Therapy

Plasma samples from 186 NSCLC patients enrolled in the BPI‐7711 phase I (NCT03386955) and phase IIa (NCT03812809) trials were analyzed. All patients had locally advanced or metastatic/recurrent disease harboring EGFR T790M mutations and had progressed on first‐ or second‐generation EGFR‐TKI therapy or presented with T790M at diagnosis.

Blood was collected in EDTA tubes and centrifuged at 16,000 g for 10 min at 4°C. Isolated plasma was stored at −80°C until analysis. Treatment response was evaluated using Response Evaluation Criteria in Solid Tumours (RECIST v1.1), and patients were classified as responders (complete or partial response) or non‐responders (stable or progressive disease). All procedures complied with the Declaration of Helsinki and were approved by the institutional ethics committee.

### 4.12 Survival Analysis

Survival analyses were conducted using the survival package in R. Optimal cutoffs for gene expression or cell infiltration were determined using maxstat test based on maximally selected rank statistics. Kaplan–Meier curves were generated, and group differences were assessed using the log‐rank test.

### 4.13 Pathological Annotation of Spatial Transcriptomics Samples

Details of spatial transcriptomics sequencing and CNV‐based tumor cell identification are provided in the supplementary materials. Each Visium spot was independently evaluated by two pathologists and assigned to histological categories, including normal epithelium, tumor, fibroblast, endothelial, or immune cells. Classification required at least 50% coverage by a given cell type.

### 4.14 Clustering of Spatial Transcriptomics Data

Gene–spot count matrices were processed using Seurat. Data normalization was performed with SCTransform, followed by identification of variable features and clustering using FindVariableFeatures, FindNeighbors, and FindClusters.

### 4.15 CARD Deconvolution

Cellular composition of spatial transcriptomics data was estimated using the CARD R package. Single‐cell reference profiles for six major cell types were integrated with spatial data using createCARDObject. Deconvolution was performed with CARD_deconvolution, and cell‐type proportions were visualized using CARD.visualize.pie.

### 4.16 ORIENT‐3 Cohort

The ORIENT‐3 phase III trial was conducted across 39 centers in China with ethics approval and informed consent from all participants (NCT03150875). Overall survival was the primary endpoint. Patients receiving anti‐PD‐1/PD‐L1 therapy before progression were excluded from the docetaxel arm analysis. Among 157 sequenced patients, 110 had qualified archival FFPE tumor samples with validated RNA sequencing data (61 sintilimab, 49 docetaxel) and were included in downstream analyses. RNA was extracted using the RNeasy FFPE Kit (Qiagen).

### 4.17 Cell–Cell Communication Analysis

Intercellular communication was inferred using CellChat. Signaling interactions were analyzed with CellChatDB.human, and the functional roles of cell populations were assessed using netAnalysis_signalingRole_scatter.

### 4.18 Statistical Analysis

Comparisons between two groups were performed using the Mann–Whitney U test. Associations were evaluated using Spearman correlation. All tests were two‐sided, and p < 0.05 was considered statistically significant. Data processing and visualization were conducted in R (version 4.1.0).

## Author Contributions

Professor YKS, XHH, and PYX designed and supervised the research. GYF, LT, TJX, LL, and SY collected the patient samples and clinical data. GYF, LT, and TJX performed bioinformatic and statistical analysis of the data. GYF, LT, and MWY wrote the manuscript. YKS, XHH, and PYX revised the manuscript. All authors have reviewed the manuscript and approved the final version for publication.

## Funding

National Science and Technology Major Project for Key New Drug Development [2017ZX09304015, 2019ZX09201‐002] and the National High Level Hospital Clinical Research Funding (2022‐PUMCH‐B‐033).

## Conflicts of Interest

The authors declare no conflicts of interest.

## Supporting information




**Supporting File**: advs74390‐sup‐0001‐SuppMat.pdf.

## Data Availability

The data that support the findings of this study are available from the corresponding author upon reasonable request.;
