# Supplementary material for: DSG2+ Cancer Stem Cells Co‐Located With FAP+ Myofibroblasts in the Tumor Boundary That Determines the Efficacy of Immunotherapy in Non‐Small Cell Lung Cancer
Source: Adv Sci (Weinh). 2026 Feb 15;13(23):e14543. doi: 10.1002/advs.202514543 (PMC13104146; doi:10.1002/advs.202514543)
Supplement: Supplementary file 1 — Supporting File: advs74390‐sup‐0001‐SuppMat.pdf. [file ADVS-13-e14543-s001.pdf]

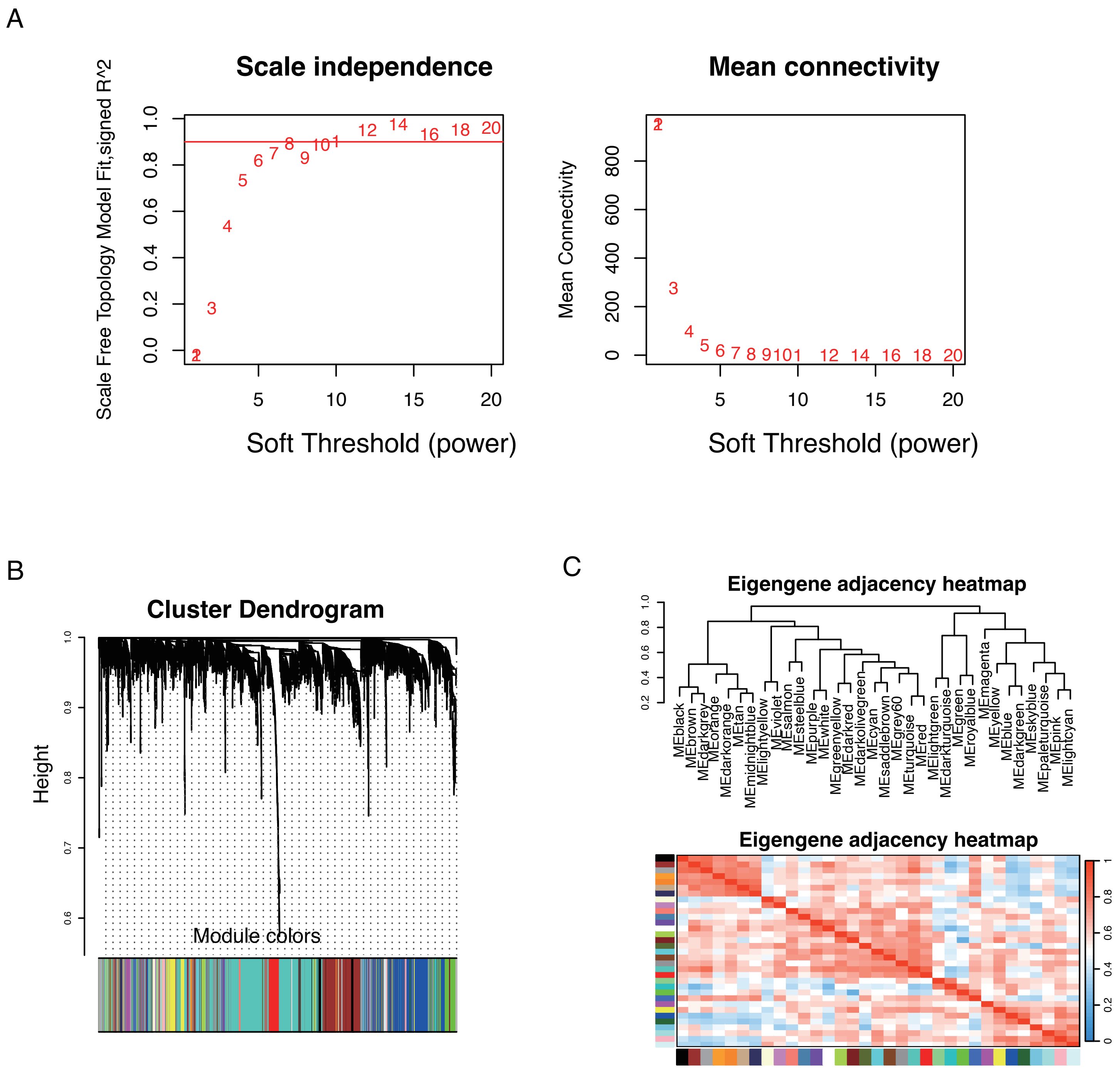

Figure S1. Identification of the cancer stem cells (CSC) signature using weighted gene co-expression network analysis (WGCNA). (C) Construction of a scale-free topology model with a soft thresholding power set to 7. (D) Hierarchical cluster tree revealing a total of 34 modules identified by WGCNA. (E) Heatmap showing the intercorrelation of each module.

Table S1. The list of genes in the CSC signature.

DSG2	OLA1	PGRMC1	SUDS3	HEBP2
KRT19	HINT2	ODC1	CCT2	EI24
TACSTD2	CPNE3	COA3	METTL5	NIPSNAP1
EPCAM	DSC2	TOMM20	MRPS18A	DKC1
KRT8	PRDX6	CCDC58	PDHA1	CCT4
KRT18	CCT6A	CCT3	RPL7L1	NDUFB9
ATP1B1	CBX1	KDM5B	HTATSF1	GLO1
S100A16	SLC39A4	NIFK	FDPS	
DMKN	NPM3	ADI1	FDFT1	
KLF5	UBAP2	MRPL24	NME1	
KRTCAP3	DPY30	C1orf35	PSMD12	
EGFR	ADSS	NDUFAF8	CYC1	
ID1	NIPSNAP2	MRPS12	RBFOX2	
TRIM2	ARPC1A	TFG	ENSA	
SPTSSA	JPT1	ECI1	WDR43	
LAPTM4B	LDHB	NSD3	BOLA3	
SINHCAF	MZT2B	CENPX	EIF6	
STOML2	SNRPD1	TMEM141	EBP	
TSTD1	FBL	COIL	SLC38A1	
ATP8B1	MRPL51	STRAP	UQCC3	
MRPL14	ATP5MC1	MZT2A	PAFAH1B3	
NAXE	PDCD5	PAICS	SF3B6	
HMGA1	SEM1	SNRPG	TXNDC17	
AHCY	KRT10	MRPS35	COQ4	
NET1	CISD1	NDUFS6	FAM136A	
BCL7A	CCDC34	MRPS10	YWHAQ	
ACTL6A	ILF2	PPA1	PSMB7	
PSMB5	IARS2	PRKDC	PRPF4	
PSMD11	C1QBP	SLC25A5	SNRPE	
SIGMAR1	SRPK1	SLC25A4	MRPL12	

Table S2: The The detailed quality information of ST samples.

sample	age	gender	stage	Mean Reads per Spot	Mean Reads Under Tissue per Spot	Fraction of Spots Under Tissue	Reads Mapped to Probe Set	Reads Mapped Confidently to Probe Set	Fraction Reads in Spots Under Tissue	Median Genes per Spot	Median UMI Counts per Spot	Genes Detected
p1	57	male	I	71070.03	70253.86	1	0.98	0.98	1	4566.5	10002	18040
p2	49	female	II	74092.74	72944.5	0.94	0.99	0.98	0.99	7138.5	30512.5	18059
p3	67	female	III	64385.89	63747	0.99	0.99	0.99	1	7729.5	38078.5	18047
p4	59	male	II	69052.37	68482.48	1	0.99	0.98	1	5697.5	14317	18062
p5	70	male	I	70420.55	69833.25	1	0.99	0.85	1	3544.5	8679.5	18072
p6	63	female	IV	72496.57	71287.92	0.9	0.99	0.98	0.99	5199.5	11014	18050

Table S3. The curated marker genes of CAF.

vCAF	CCL8, GJA4, MHY11, MCAM, RGS5, IL-6
myCAF	COL1A1, COL1A2, COL3A1, COL5A1, COL5A2, COL6A3, DCN FN1, POSTN
apCAF	CD74, HLA-DRA, HLA-DRB1,CCL21,CXCL12
mesCAF	KRT19, KRT8, SAAl, SLPI
iCAF	CXCL1, C3, C7, FBLN1, IGF1, IGFBP6, CCL19
adipoCAF	APOA2, FABP1, FABP4, FRZB, GPX3

Table S4. The list of marker genes of myCAF.

POSTN	FKBP10	DYNLT1	COL11A1	MMP2	RNU6-6P
AEBP1	C1QB	LAPTM5	COL6A1	DIO2	BHLHE41
INHBA	CNIH3	NOX4	NTM	NMT1	EVA1A
COL1A1	PCOLCE	NDUFC2	CHN1	TMED9	AIF1
MMP12	ARF4	RHOBTB3	PRRX1	RCN3	SDC1
MMP9	LMAN1	IL24	TCEAL9	ETS1	ERRFI1
FN1	TYMP	ANGPTL2	MMP14	SELENOK	KLF6
SLC44A1	IFI27	ACTN1	C5orf46	RIN2	TMED2
COL5A1	PPFIBP1	ANTXR1	GUCY1A1	REEP3	S100A2
SFRP2	PLPP4	WDR1	RPS29	RPS27L	FAM32A
LUM	THBS2	TBCA	PRSS23	CERCAM	C12orf75
EMP1	RN7SL2	FAM198B	FADS2	GSTO1	IGF2
OLFML3	AC134312.5	LPAR6	PDXDC1	PLAU	PTPRE
SPARC	FCER1G	PLXNC1	DAB2	MFGE8	MME
GREM1	IGF2BP2	PIP5K1C	SNAI2	COL12A1	SOD2
CBX3	HLA-E	BCAT1	KRT16	HAS2	MXRA5
CTSK	COL10A1	FBXO32	SLC16A3	TBX3	P3H1
GRP	FSTL1	GLT8D2	CD82	SLC1A3	SNHG5
FAP	SOX4	FKBP9	POU2F2	ENO1	ANPEP
MT-ATP8	MT-ND6	MCFD2	ITM2C	SLK	CDKL5
CTHRC1	GPR68	SRPX2	ANXA2	CTSL	BICC1
SUGCT	TMEM258	CHI3L1	BGN	LEPROT	RNY3
COL6A3	GPM6B	PLOD2	SPP1	CTGF	UQCR11
MMP12	APOE	SUSD6	LY6E	SLAMF8	PSMC1
MMP11	TCF4	MYL12A	FBLN2	C1QA	NREP
SERPINH1	LMO4	MICAL2	WNT5A	RPL23A	SAT1
TNFAIP6	EIF2S2	AP2B1	MORF4L1	AL365357.1	ATP6AP2
HLA-B	PLAUR	C4orf3	MMP1	TCEAL8	PDLIM4
ISLR	LAMA4	WNT2	ARID5B	COL6A2	HOXB-AS1
DCN	STEAP1	RAI14			

Methods

Cell culture and DSG2 knockdown

Human NSCLC cell lines A549 and HCC827 were obtained from the American Type Culture Collection (ATCC, Manassas, VA). Cells were cultured in RPMI-1640 medium (Corning, #15-041-CV) supplemented with 10% fetal bovine serum (FBS; HyClone, #SH30084), 100 U/mL penicillin, and 100 μg/mL streptomycin (Gibco, #15140122), and maintained at 37 °C in a humidified incubator with 5% CO_2. The identities of all cell lines were regularly confirmed by short tandem repeat (STR) profiling.

DSG2 knockdown was achieved using short hairpin RNA (shRNA). Briefly, nonsense control and DSG2-targeting shRNA plasmids (5′-CACCGCTTCTCCTGATCTGCTTTAACGAATTAAAGCAGATCAGGAGAA GC-3′) were synthesized by GeneChem (Shanghai, China). Cells were infected with lentiviral particles encoding control or DSG2-targeting shRNAs, followed by selection according to the manufacturer's instructions. Cells were harvested 48–72 h after transfection for subsequent molecular and functional analyses. Knockdown efficiency was validated at the protein level by Western blot analysis prior to downstream experiments.

Analysis of CSC-associated marker expression

To evaluate molecular changes associated with stemness, whole-cell lysates were prepared using RIPA buffer supplemented with protease and phosphatase inhibitor cocktails. Protein concentrations were determined using a BCA protein assay kit. Equal amounts of total protein (20–30 μg) were separated by SDS–PAGE and transferred onto PVDF membranes. After blocking with 5% non-fat milk, membranes were incubated with primary antibodies against CSC-associated transcription factors, including OCT4 (Abcam, ab18976), SOX2 (Abcam, ab97959), NANOG (Abcam, ab80892), and c-MYC (Abcam, ab32072), as well as CSC surface markers CD133 (Abcam, ab216323), CD44 (Abcam, ab189524), and ALDH1A1 (Abcam, ab52492). GAPDH (Abcam, ab8245) was used as a loading control. All primary antibodies were applied according to the manufacturers' recommended dilutions. Following incubation with appropriate HRP-conjugated secondary antibodies , protein signals were detected using enhanced chemiluminescence reagents. Band intensities were quantified using ImageJ software and normalized to the corresponding loading controls.

Self-renewal assays

CSC self-renewal capacity was assessed using single-cell clonogenic assays and tumorsphere formation assays. For single-cell clonogenic assays, tumor cells were

dissociated into single-cell suspensions and seeded at low density (200–500 cells per well) in 6-well plates under adherent culture conditions. Cells were cultured for 10–14 days with medium refreshed every 3–4 days. Colonies were then fixed with 4% paraformaldehyde, stained with crystal violet, and colonies containing more than 50 cells were counted. Clonogenic efficiency was calculated as the percentage of seeded cells that formed colonies. For tumorsphere formation assays, cells were seeded at a density of 500–1,000 cells per well in ultra-low attachment plates and cultured in serum-free sphere-forming medium consisting of DMEM/F12 supplemented with B27(1:50, Invitrogen), epidermal growth factor (EGF, 20 ng/mL, Peprotech), and basic fibroblast growth factor (bFGF, 20 ng/mL, Peprotech). After 7–10 days of culture, tumorspheres were imaged using phase-contrast microscopy. Sphere number and sphere diameter were quantified, and only spheres with a diameter greater than 50 μm were included in the analysis.

Cell proliferation and invasion assays

Cell proliferation was assessed using the Cell Counting Kit-8 (CCK-8; Dojindo, Japan) according to the manufacturer's instructions. Tumor cells were seeded into 96-well plates at a density of $1–3 \times 10^3$ cells per well. At the indicated time points, CCK-8 reagent was added, and absorbance at 450 nm was measured using a microplate reader to evaluate relative cell viability. Cell invasive capacity was evaluated using Matrigel-coated Transwell invasion assays with 8-μm pore size inserts (Corning, USA). Briefly, tumor cells ($5–10 \times 10^4$ cells) were suspended in serum-free medium and seeded into the upper chamber, while medium containing 10% fetal bovine serum (FBS; HyClone, #SH30084) was added to the lower chamber as a chemoattractant. After 24–48 h of incubation, non-invading cells were removed from the upper surface of the membrane. Invading cells on the lower surface were fixed with paraformaldehyde, stained with crystal violet, and quantified under a microscope.

Isolation of DSG2^high CSC-enriched tumor cells

A549 and HCC827 tumor cells were dissociated into single-cell suspensions using a non-enzymatic or mild enzymatic dissociation method and resuspended in ice-cold PBS containing 1–2% FBS. Cells were passed through a 40-μm cell strainer to remove aggregates prior to staining. Single-cell suspensions were incubated with a fluorochrome-conjugated anti-DSG2 antibody (R&D Systems, FAB947P) for fluorescence-activated cell sorting (FACS,) according to the manufacturer's instructions. Appropriate isotype controls were included to define background fluorescence. Live cells were gated based on forward and side scatter properties and exclusion of dead cells. DSG2^high and DSG2^low populations were defined as the top and bottom 10% fractions of DSG2-expressing cells, respectively, based on fluorescence intensity. Sorted cells were collected into complete culture medium and allowed to recover under standard culture conditions prior to downstream analyses. The CSC-enriched nature of DSG2^high cells was validated by functional assays, including enhanced tumorsphere formation and increased single-cell clonogenic

capacity compared with DSG2^low cells. DSG2^high CSC-enriched tumor cells were subsequently used for all co-culture experiments.

Generation and characterization of FAP$^+$ myCAFs

Primary lung fibroblasts from the American Type Culture Collection (ATCC, Manassas, VA) were cultured under standard conditions and differentiated into myofibroblastic cancer-associated fibroblasts (myCAFs) by treatment with transforming growth factor–β (TGF-β1, Peprotech) (10 ng/mL) for 72 h to induce myCAF differentiation. Briefly, fibroblasts were cultured in complete medium supplemented with recombinant TGF-β at the indicated concentration for the indicated duration to induce myCAF differentiation. Successful induction of FAP$^+$ myCAFs was confirmed by increased expression of fibroblast activation protein (FAP，Abcam, ab207178), ACTA2 (α-SMA, Abcam, ab5694), and matrix metalloproteinases (MMP9 Abcam, ab38898 and MMP12 Abcam, ab52897), as assessed by gene expression and/or protein expression analyses. Fibroblasts cultured in the absence of TGF-β were used as control fibroblasts in subsequent co-culture experiments.

Transwell co-culture assays

To investigate paracrine interactions between tumor cells and fibroblasts, Transwell co-culture assays were performed using inserts with a semi-permeable membrane that prevents direct cell–cell contact. We utilized 6-well Transwell chambers with 0.4 μm pores (PTHT06H48, Corning). DSG2^high CSC-enriched tumor cells were seeded in the lower chamber, while control fibroblasts or FAP$^+$ myCAFs were seeded in the upper chamber. Tumor cells cultured alone served as an additional control condition. Four experimental conditions were analyzed: (1) DSG2^high tumor cells cultured alone; (2) DSG2^high tumor cells co-cultured with control fibroblasts; (3) DSG2^high tumor cells co-cultured with FAP$^+$ myCAFs; and (4) DSG2^high tumor cells co-cultured with FAP$^+$ myCAFs in the presence of the broad-spectrum matrix metalloproteinase inhibitor GM6001. For MMP inhibition experiments, GM6001 (Sigma-Aldrich) was added to the co-culture system at the indicated concentration 10 μM previously reported to inhibit MMP activity without affecting cell viability.throughout the co-culture period. Co-culture was maintained for 72 hours before tumor cells were harvested for downstream analyses.

Assessment of CSC-associated phenotypes

Following co-culture, tumor cells were harvested and subjected to functional and molecular analyses. CSC-associated properties were evaluated by examining the expression of CSC markers and EMT-related proteins, as well as by functional self-renewal assays. Self-renewal capacity was assessed using single-cell clonogenic assays and tumorsphere formation assays, with sphere number and size quantified as indicators of stemness. Tumor cell proliferative capacity was assessed using CCK-8

assays. Migratory and invasive capacities were evaluated using Transwell migration and Matrigel invasion assays according to standard protocols.

Statistical analysis

All experiments were performed with at least three independent biological replicates. Quantitative data are presented as mean ± standard deviation (SD). Statistical analyses were performed using appropriate statistical tests as specified in the figure legends, and $p < 0.05$ was considered statistically significant.

Target	Antibody	Vendor	Catalog No.	Host	Application	Expected MW
**DSG2**	Anti-Desmoglein 2	Abcam	**ab150372**	Rabbit mAb	WB, IF	~122 kDa
**OCT4 (POU5F1)**	Anti-OCT4	Abcam	**ab18976**	Rabbit mAb	WB, IF, IHC	~39–45 kDa
**SOX2**	Anti-SOX2	Abcam	**ab97959**	Rabbit mAb	WB, IF, IHC	~34 kDa
**NANOG**	Anti-NANOG	Abcam	**ab80892**	Rabbit pAb	WB, IF	~35 kDa
**c-MYC**	Anti-c-MYC	Abcam	**ab32072**	Rabbit mAb	WB, IF, IHC	~57–64 kDa
**CD133 (PROM1)**	Anti-CD133	Abcam	**ab216323**	Rabbit mAb	WB, IF	~120 kDa
**CD44**	Anti-CD44	Abcam	**ab189524**	Rabbit mAb	WB, IF, IHC	~85–90 kDa
**ALDH1A1**	Anti-ALDH1A1	Abcam	**ab52492**	Rabbit mAb	WB, IF	~55 kDa
**FAP**	Anti-FAP	Abcam	**ab53066**	Rabbit mAb	WB, IF, IHC	~95 kDa
**α-SMA (ACTA2)**	Anti-α-SMA	Abcam	**ab32575**	Mouse mAb	WB, IF, IHC	~42 kDa
**MMP9**	Anti-MMP9	Abcam	**ab76003**	Rabbit mAb	WB	~92 kDa
**MMP12**	Anti-MMP12	Abcam	**ab52897**	Rabbit mAb	WB	~54 kDa
**GAPDH**	Anti-GAPDH	Abcam	**ab8245**	Mouse mAb	WB	~36 kDa

PAPER NAME

20260127123658320008.docx

WORD COUNT

5184 Words

CHARACTER COUNT

33057 Characters

PAGE COUNT

20 Pages

FILE SIZE

80.9KB

SUBMISSION DATE

Jan 27, 2026 8:38 PM GMT+8

REPORT DATE

Jan 27, 2026 8:39 PM GMT+8

18% Overall Similarity

The combined total of all matches, including overlapping sources, for each database.

- 15% Internet database
- 16% Publications database
- Crossref database
- Crossref Posted Content database

Excluded from Similarity Report

- Bibliographic material
- Quoted material

Abstract

Lung cancer remains a leading cause of cancer-related death owing to its aggressiveness and marked heterogeneity. Emerging evidence suggests that cancer stem cells (CSCs) exist as a dynamic and plastic population capable of transitioning between stem-like and differentiated states, yet their transcriptional features and spatial organization are incompletely understood. We integrated single-cell RNA sequencing of 92,820 cells from 40 tumors with spatial transcriptomics of 28,712 spots from six samples to define CSC-associated programs in non-small cell lung cancer (NSCLC). Weighted gene co-expression network analysis identified a 127-gene CSC signature enriched for stemness pathways, with DSG2 as the most prominent marker. Elevated DSG2 expression was associated with chemotherapy resistance in the ORIENT-3 trial and predicted response to EGFR-TKI therapy based on serum proteomic profiling from the BPI-7711 study.Spatial mapping revealed preferential localization of DSG2+CSCs at tumor margins, where they co-localized with FAP+myofibroblasts (myCAFs). Multiplex immunofluorescence confirmed this niche and showed that myCAFs expressed MMP9 and MMP12, consistent with enhanced epithelial–mesenchymal transition and CSC maintenance. Both DSG2+ CSCs and FAP+myCAFs correlated with poor immunotherapy outcomes. Functionally, myCAF co-culture enhanced CSC-associated phenotypes in DSG2^high tumor cells, an effect that was partially attenuated by pharmacological inhibition of MMP activity, supporting an MMP-dependent paracrine interaction. Both DSG2+CSCs and FAP+myCAFs correlated with poor immunotherapy outcomes. Together, these findings define a robust CSC signature and uncover a spatially organized DSG2+ CSC–myCAF niche that contributes to therapeutic resistance, providing potential biomarkers and targets for precision treatment in NSCLC.

Keywords: cancer stem cells, lung cancer, single-cell RNA sequencing, spatial transcriptomics, immunotherapy, EGFR-TKI therapy

Introduction

Lung cancer remains one of the most lethal malignancies worldwide, characterized by

pronounced biological heterogeneity, rapid progression, and poor long-term outcomes[illegible]. Non-small cell lung cancer (NSCLC) constitutes the majority of cases[3], yet survival remains unsatisfactory despite advances in targeted therapy, immunotherapy, and early detection strategies. Across all stages, the 5-year survival rate is approximately 25% and falls below 10% in metastatic disease[4]. Even patients undergoing curative-intent surgery frequently relapse, and although many individuals initially respond to molecularly targeted agents or immune checkpoint blockade, durable benefit is limited by the near-universal emergence of therapeutic resistance[5–8].

Increasing evidence suggests that these clinical challenges are closely linked to tumor cell plasticity and microenvironmental influences[9]. Cancer stem cells (CSCs) have been proposed as a key driver of tumor initiation, progression, and recurrence[10–12]. Traditionally, tumors were viewed as hierarchically organized, with a rare stem-like population capable of self-renewal and differentiation. More recently, however, the phenotype plasticity model has reframed CSCs as a dynamic state rather than a fixed subset, in which cancer cells can reversibly transition between stem-like and non-stem-like phenotypes[13,14]. This flexibility enables tumor cells to regenerate stem-like populations after therapy and contributes to disease persistence[15,16].

Most prior studies have relied on surface markers and functional assays, such as cell sorting and xenotransplantation, to identify CSCs[17–22]. Although markers including CD13, CD24, EPCAM, CD44, and CD133 have been widely used, they are not tumor-specific and provide limited insight into the transcriptional continuum underlying stemness[23–25]. Consequently, the molecular programs that define CSC states and their spatial organization within tumors remain incompletely characterized.

CSCs also actively remodel the tumor microenvironment (TME) to promote survival[26,27]. By suppressing cytotoxic immune responses, expressing immune checkpoint molecules, and enhancing angiogenesis and tissue remodeling, CSCs foster an immunosuppressive niche that protects them from therapy[28]. In turn, stromal

and immune components of the TME reinforce stem-like traits, creating reciprocal interactions that sustain resistance to both targeted therapy and immunotherapy[29,30]. Dissecting this bidirectional crosstalk is therefore essential for improving treatment efficacy. Spatial transcriptomics provides an opportunity to address these gaps by simultaneously resolving gene expression and tissue architecture. This approach enables precise mapping of CSC-enriched regions, identification of neighboring stromal or immune populations, and characterization of cell－cell interactions within defined niches[12]. Leveraging Spatial transcriptomics, together with single-cell analyses, allows a more comprehensive understanding of how CSCs are organized and supported within the lung cancer microenvironment[31,32].

We hypothesized that CSCs in NSCLC display distinct transcriptional programs and spatial organization that drive tumor progression and therapeutic resistance. To investigate this, we integrated single-cell RNA sequencing from 92,820 cells across 40 tumors with spatial transcriptomics from 28,712 spots across six samples. This approach enabled construction of a CSC gene signature, mapping of CSC localization within the tumor microenvironment, and assessment of associations between CSC markers and treatment response, including chemotherapy, targeted therapy, and immunotherapy. Together, these analyses define the molecular and spatial features of CSCs and clarify their contribution to disease progression and therapy resistance.

Results

The signature of CSC was identified using weighted gene co-expression network analysis

The workflow was depicted in Figure 1. Single-cell RNA sequencing data from 42 NSCLC tumors in GSE148071 (107,761 cells) were analyzed to define stem-like tumor populations[33]. Clustering resolved six major cell types, including epithelial, myeloid, fibroblast, endothelial, T, and B cells. We utilized a rigorous approach involving the integration of weighted gene co-expression network analysis (WGCNA) and the Metacell algorithm[34,35].

Single-cell RNA sequencing data is inherently sparse and noisy, which can mask true gene-gene correlations and confound the analysis of co-expression networks. Additionally, we observed considerable variation among malignant cells, both within individual patients and between different patients. While malignant cells displayed clear groupings based on their sample source, non-malignant cells showed limited noticeable differences among individuals (Figure 2A). This observation indicates that the heterogeneity of tumor cells is primarily due to the tumor cells themselves rather than the samples. The heterogeneity among cancer cells in our dataset necessitated modifying the traditional WGCNA workflow commonly applied to bulk transcriptomic data. We employed the Metacell algorithm to partition cells into homogenous groups. After quality control, a total of 50,275 malignant cells were selected and divided into 498 metacells (Figure 2B).

Because single-cell data are sparse and heterogeneous, direct co-expression analysis can be unreliable. We also observed substantial transcriptional variability among malignant cells both within and across patients, whereas non-malignant populations showed relatively limited inter-individual differences (Figure 2A). To address this heterogeneity and improve signal stability, malignant cells were aggregated into transcriptionally similar metacells. After quality control, 50,275 tumor cells were grouped into 498 metacells (Figure 2B), providing a refined representation of gene expression patterns.

Co-expression networks were then constructed using the 5,000 most variable genes across metacells. A soft-thresholding power of 7 was selected to approximate scale-free topology (Figure S1A), resulting in 34 gene modules (Figure S1B). Correlating module eigengenes with CytoTRACE scores identified the turquoise module as most strongly associated with stemness (Figure 2C). Overlap between genes in this module and malignant-cell markers (average log fold change > 0.5, $P < 0.05$) produced a set of 127 tumor-enriched genes, which we defined as the CSC

signature (Figure 2D, Table S1). This signature included several well-established stemness-associated genes, such as EPCAM and EGFR. CSC activity quantified using AUCell showed consistent agreement with CytoTRACE scores (correlation ~0.8) and was reproducible across three independent NSCLC datasets (GSE127465, GSE143423, and EMTAB6149) (Figure 2E).

DSG2 had negative roles in chemotherapy and EGFR-TKI therapy

Consequently, we examined the role of CSCs in predicting the treatment effectiveness for patients receiving anti-tumor therapies. We integrated large-scale transcriptomics data from the ORIENT-3 clinical trial (NCT03150875)[36]. The dataset included 49 patients with NSCLC who received chemotherapy and 61 NSCLC patients who received immunotherapy. Upon initial examination of the chemotherapy subset, we discovered that NSCLC patients with higher CSC score or greater levels of DSG2 had shorter OS times (Figure 3A). DSG2, a CSC marker, exhibited elevated expression levels in both non-responders (NR) and responders (R) (Figure 3B). Survival analysis demonstrated that higher DSG2 expression was significantly associated with worse clinical outcomes, with a hazard ratio (HR) of 1.35 ($p = 0.0044$). Receiver operating characteristic (ROC) analysis further supported the predictive value of DSG2 for chemotherapy response, yielding an area under the curve (AUC) of 0.812 ($p = 0.015$).

We then conducted a thorough analysis using multi-omics data to investigate the clinical implications of CSCs in NSCLC patients who received EGFR-TKI therapy. Initially, we collected single-cell data from 49 clinical biopsies of 30 patients with metastatic lung cancer before and during targeted therapy[37]. Using the AUCell R program, we computed the CSC score in each tumor cell and observed that the expression pattern of DSG2 closely resembled the CSC score, suggesting that DSG2 accurately captures stemness characteristics (Figure 3C). Both the CSC score and DSG2 exhibited increased expression in NR patients (Figure 3D). Additionally, we acquired mRNA data from a group of 44 patients who received targeted therapy as part of the IMPACT research (https://src.gisapps.org/OncoSG/). The CSC score and

DSG2 marker showed a noteworthy negative correlation with poorer clinical outcomes in individuals treated with EGFR-TKIs (Figures 3E, 3F).

We further examined serum proteomic and clinical data from a prospective cohort. This group consisted of 186 patients with locally advanced or metastatic NSCLC in BPI-7711 clinical trail (NCT03386955)[36]. Quantification of 521 proteins in patient plasma samples obtained at baseline was performed using mass spectrometry. Among the 186 patients, 57 (30.65%) did not experience clinical improvement, while 129 (69.35%) derived benefits from the medication. Out of the 521 proteins in the proteomic data, 11 proteins were present in CSC signature. DSG2 showed the most significant increase in expression of the 11 proteins in NR compared to R (Figure 3G). DSG2 also had a notable negative correlation with poorer clinical outcomes in patients treated with EGFR-TKI (Figure 3H). Elevated circulating DSG2 levels were strongly associated with inferior therapeutic outcomes, with an HR of 2.17 (p = 0.00014). ROC analysis demonstrated robust discriminatory performance, with an AUC of 0.913 (p = 0.00047). These findings suggest that DSG2 has potential as a biomarker for predicting treatment outcomes.

DSG2+ CSC co-located with FAP+ myCAF in the tumor boundary

To elucidate the spatial organization associated with CSCs, we utilized cell type deconvolution through the CARD R package[38]. This approach integrates cell-type-specific expression data from single-cell RNA sequencing to infer cellular composition across tissue regions. Initially, we categorized spots into six primary cell types—epithelial cells, myeloid cells, fibroblasts, endothelial cells, T cells, and B cells (Figure 5A). These results were consistent with HE pathological annotations, revealing fibroblasts as the predominant cell type infiltrating the tumor area, followed by myeloid cells and endothelial cells. Immune cells such as B cells and T cells were sparsely distributed within the tumor, with fibroblasts prominently surrounding the tumor, potentially supporting the maintenance of CSCs and suggesting a pro-tumor role.

Given the diverse functions of cancer-associated fibroblasts (CAFs) in the TME, we investigated specific subtypes of CAFs present in the tumor region. Selected markers for six CAF subtypes—myofibroblasts (myCAF), inflammatory CAFs (iCAFs), antigen-presenting CAFs (apCAFs), vascular CAFs (vCAFs), mesothelial CAFs (mesCAF), and adipogenic CAFs (adipoCAF)—were analyzed (Table S3). Our findings indicated that myCAF subtype showed exclusive infiltration within the tumor area, while the other subtypes were more abundant in neighboring normal tissue (Figure 5B).

We further explored the correlation between DSG2 expression and the myCAF signature, revealing a high correlation coefficient (R=0.67, p<0.001) (Figure 5C). Among the typical markers of myCAF, FAP exhibited the strongest correlation with DSG2 (R=0.42, p<0.001) (Figure 5C). We labeled the gene expression of DSG2 and FAP greater than zero as DSG2+FAP+ spots, indicating the co-location of DSG2+ tumor cells and FAP+ CAF. Additionally, spots with either DSG2 or FAP expression greater than zero were labeled as DSG2+ spots or FAP+ spots, respectively. The distribution of these three types of spots in Figure 5D shows that DSG2+FAP+ spots are the majority among the spots expressing DSG2 and FAP. The DSG2+ spots and FAP+ spots are situated around the DSG2+FAP+ spots, highlighting a strong co-localization pattern of DSG2+ tumor cells and FAP+ CAF (Figure 5D). Across all samples, DSG2^{+}FAP+spots accounted for 78.24% – 91.72% of the DSG2- or FAP-expressing spots, indicating that the majority of DSG2- and FAP-positive spots exhibit concurrent expression of both genes. Continual observation across samples confirmed co-localization of DSG2+ CSCs and FAP+ myCAFs, suggesting physical interaction within the niche (Figure 5D). To validate these findings at the protein level, multiplex immunofluorescence analysis was performed on 20 NSCLC tumor samples. Using panCK to identify tumor cells, DSG2 to identify CSCs, and FAP to identify myCAFs, we confirmed a robust spatial relationship between DSG2+ CSCs and FAP+myCAFs at the tumor boundary, implying potential communication between

these cell types (Figure 5E). These results underscore the intricate interplay between CSCs and specific CAF subtypes, particularly myCAFs, in shaping the tumor microenvironment and influencing disease progression.

DSG2+ CSC and FAP+ myCAF had negative roles in immunotherapy

To precisely delineate the characteristics of FAP+ myCAF, we conducted a comprehensive study examining different CAFs at the single-cell level using the GSE148071 dataset. Initially, we classified fibroblasts (n=3928) into six distinct subclusters: myCAF, iCAF, apCAF, adipoCAF, vCAF, and mesCAF (Figure 6A). Notably, myCAF emerged with unique functional attributes compared to other fibroblast subtypes, characterized by high expression of FAP. Moreover, myCAF exhibited elevated levels of metalloproteinases such as MMP9 and MMP12, crucial for extracellular matrix remodeling and immune response modulation (Figure 6B). Differential gene expression analysis revealed that myCAF was enriched in pathways related to EMT, angiogenesis, TGFB signaling, and glycolysis, while immune-related pathways like complement activation and humoral immune response were notably suppressed in myCAF (Figure 6C). These findings suggest that myCAF may play a role in promoting EMT and suppressing immune responses in NSCLC.

Examining data from the TCGA cohort further underscored the clinical relevance of FAP+ myCAF. We observed a significant increase in FAP+ myCAF infiltration in tumor samples compared to adjacent healthy tissues, with higher levels correlating with advanced stages of NSCLC and poorer OS outcomes in NSCLC patients (Figure 6D, 6E, and 6F). Given the pivotal roles of CSCs and myCAF in influencing treatment responses, we investigated the predictive potential of DSG2+ CSC and FAP+ myCAF in patients undergoing immunotherapy using data from the ORIENT-3 clinical trail[39]. Analysis revealed that individuals with higher levels of CSCs and myCAF were associated with poorer responses to immunotherapy (Figure 6G, 6H, 6I, and 6J). These findings underscore the importance of CSCs and myCAF as potential biomarkers for predicting immunotherapy outcomes in NSCLC patients, highlighting

their relevance in clinical decision-making.

Cell-cell communication between DSG2+ CSC and FAP+ myCAF

In our study, we investigated the interactions between DSG2+ CSCs and FAP+ myCAFs, focusing on their co-location at the boundary area of the tumor. Recognizing precise interactions between DSG2+ CSCs and FAP+ myCAFs was challenging due to the limitation of the 10X Genomics Visium platform, which accommodates 1-10 cells per spot. Therefore, we initially evaluated their putative crosstalk at the single-cell level. Our analysis identified the MK and SPP1 signaling pathways as key mediators of communication between these cell types (Figure 7A). In the self-communication of DSG2+ CSCs, SPP1 interacted with CD44, ITGAV, and ITGB1. Midkine (MDK), a key ligand of the MK signaling pathway, has been implicated in supporting cancer progression in various contexts. Our findings indicated that MDK-SDC1, MDK-SDC2, MDK-PTPRZ1, and MDK-(ITGA6+ITGB1) actively participated in the self-communication of DSG2+ CSCs. Furthermore, MDK released from FAP+ myCAFs activated nucleolin (NCL) in DSG2+ CSCs. NCL, a major nucleolar protein of growing cells, also functions as a cell surface receptor, shuttling between the cytoplasm and nucleus, thereby providing a mechanism for extracellular regulation of nuclear events.

Given the co-location of DSG2+ CSCs with FAP+ myCAFs at the tumor boundary, we concentrated our analysis on intercellular communication in this area. The boundary area was defined as the nearest 2-spot width region near the outermost circle of the tumor boundary line, encompassing all clusters (Figure 7B). In this region, MDK was found to activate NCL in DSG2+ CSCs, with FAP+ myCAFs releasing MDK in the N1 and N2 clusters. The MK signaling pathway exhibited the highest relative strength among all communication activities (Figure 7D). To validate our findings at the protein level, we performed multiplex immunofluorescence on 20 tumor samples from NSCLC patients, using panCK antibody to annotate tumor cells, DSG2 to annotate CSCs, and FAP to annotate myCAFs. The results demonstrated that

MDK, a secretory protein from FAP+ myCAFs, activated NCL in DSG2+ CSCs, thereby confirming the specific cell-cell interaction of this ligand-receptor pair (Figure 7E). These findings consistently supported our single-cell and spatial transcriptomics results, emphasizing the critical role of MDK's interaction with NCL on DSG2+ CSCs in the tumor boundary area.

DSG2 promotes CSC-associated functional properties and proliferative capacity in NSCLC cells

To define the functional role of DSG2 in NSCLC, DSG2 was silenced in A549 and HCC827 cells using two independent shRNAs. Immunoblotting confirmed efficient DSG2 knockdown (Figure 8A), which was accompanied by marked downregulation of stemness-associated transcription factors (OCT4, SOX2, NANOG, and MYC; Figure 8B) and canonical CSC markers (CD44, ALDH1A1, and CD133; Figure 8C).

Functionally, DSG2 depletion significantly impaired CSC-associated properties. Tumorsphere formation assays showed a pronounced reduction in sphere size and number in DSG2-silenced cells (Figure 8D), and single-cell clonogenic assays revealed markedly decreased colony-forming capacity (Figure 8E). Quantitative analyses confirmed significant reductions in plate colony numbers and relative sphere-forming efficiency in both cell lines (Figure 8G). In addition, DSG2 knockdown substantially suppressed invasive potential, as demonstrated by Transwell invasion assays and corresponding quantification (Figure 8F–G), indicating a role for DSG2 in promoting invasive CSC-associated behaviors. Cell viability assays further revealed that DSG2 depletion also reduced proliferative capacity over time (Figure 8H), suggesting that DSG2 contributes to tumor cell growth in addition to regulating CSC-associated functional properties.

Together, these data demonstrate that DSG2 is functionally required for maintaining CSC-associated molecular programs, self-renewal capacity, invasiveness, and proliferative potential in NSCLC cells.

FAP+myCAFs functionally enhance CSC-associated properties of DSG2^high tumor cells through MMP-dependent paracrine signaling

To functionally test CSC – fibroblast interactions, DSG2^high and DSG2^low tumor cell populations were isolated from A549 and HCC827 cells by fluorescence-activated cell sorting (FACS). Cells were ranked by DSG2 fluorescence intensity, and the top and bottom 10% fractions were collected as DSG2^high and DSG2^low populations, respectively. Post-sort analysis confirmed high purity of the isolated subsets in both cell lines (Figure 9A), and western blotting further validated markedly higher DSG2 protein abundance in DSG2^high cells compared with DSG2^low cells (Figure 9B).

To establish a stromal model, fibroblasts were differentiated into FAP+myCAFs, as confirmed by increased expression of FAP, ACTA2 (α-SMA), MMP9, and MMP12 (Figure 9C). DSG2^high tumor cells were then subjected to Transwell co-culture with either control fibroblasts or FAP+myCAFs, with or without the broad-spectrum MMP inhibitor GM6001. Co-culture with FAP+myCAFs substantially increased the expression of stemness-associated transcription factors (OCT4, SOX2, NANOG, and MYC) and canonical CSC markers (CD44, ALDH1A1, and CD133) in DSG2^high tumor cells, whereas control fibroblasts exerted minimal effects (Figure 9D). Importantly, GM6001 markedly attenuated the myCAF-induced upregulation of these CSC-associated markers, indicating partial dependence on MMP activity.

Consistent with these molecular changes, FAP+myCAF co-culture significantly enhanced clonogenic growth, tumorsphere formation, and invasive capacity of DSG2^high tumor cells in both A549 and HCC827 cells, and these effects were reduced by GM6001 treatment (Figure 9E – F). In parallel, CCK-8 assays showed that FAP+myCAFs also promoted the proliferative capacity of DSG2^high tumor cells, which was partially diminished upon MMP inhibition (Figure 9G). Together, these data demonstrate that FAP+myCAFs actively promote CSC-associated phenotypes and tumor cell growth in DSG2^high tumor cells through MMP-dependent paracrine

mechanisms, supporting a functional CSC–myCAF interaction beyond spatial co-localization.

Discussion

CSCs drive tumor progression, metastasis, and therapeutic resistance largely by shaping an immunosuppressive TME[40]. However, their spatial organization and functional interactions in NSCLC remain insufficiently defined. Here, we established a single-cell-derived CSC gene signature and examined their spatial association with myCAFs. We further evaluated the relationship between CSC–myCAF niches and immunotherapy outcomes, highlighting their potential value as predictive biomarkers and therapeutic targets.

DSG2, a constituent of desmosomal cell-cell adhesion structures found in epithelial tissues, plays pivotal roles in various biological processes including EMT, cell proliferation, and migration[41,42]. Recent research has highlighted its involvement in enhancing the malignant properties of stem cells by activating the Wnt/β-catenin signaling pathway. In cancers like cutaneous squamous cell carcinoma, DSG2 contributes to the formation of extracellular vesicles, impacting tumor invasion and drug resistance, and serving as a prognostic and therapeutic predictor[43]. Additionally, DSG2 promotes tumor growth, facilitates clustering of circulating tumor cells, and supports metastasis to distant organs[44]. Its interaction with hypoxia influences cancer cell dissemination in breast cancer, correlating with poor prognosis and increased recurrence risk in patients[45].

Consistent with the dynamic CSC model, DSG2 should not be viewed as an exclusive or static CSC marker. Instead, its expression likely reflects a plastic stem-like state that tumor cells can acquire or lose in response to microenvironmental cues. This interpretation is further supported by spatial transcriptomics and multiplex immunofluorescence analyses, which revealed that DSG2+ CSCs are enriched at the tumor boundary and spatially co-localized with FAP+ myofibroblasts, a niche

implicated in CSC maintenance. Moreover, immunohistochemical validation across an independent cohort demonstrated that DSG2 expression is significantly elevated in tumor epithelium compared with adjacent normal tissue, reinforcing its tumor-biased and CSC-associated nature.

Despite this, developing DSG2-targeted strategies may still be feasible for several reasons. First, many tumors show markedly elevated and/or more homogeneous DSG2 expression compared with most normal tissues, which may provide a therapeutic window, particularly in molecularly selected patients with high DSG2 expression. Second, multiple modality and engineering approaches could mitigate normal-tissue liability, including (i) antibody – drug conjugates (ADCs) or radioligand approaches that rely on differential antigen density/internalization to preferentially affect DSG2-high tumor cells, (ii) affinity-tuned antibodies/CARs or logic-gated designs to reduce recognition of low-level DSG2 in normal tissues, and (iii) tumor-restricted delivery strategies (e.g., local delivery or conditionally activated/prodrug-like formats) to further improve safety.

In the context of TME modulation by CSCs, our investigation has revealed insights into their role in tumor immunity. CAFs, prominent stromal cells in the TME, are critical in conferring resistance to therapies through diverse mechanisms[46,47]. Specifically, CAFs in NSCLC sustain CSC stemness via paracrine signaling pathways, particularly highlighted in co-culture experiments[48]. Notably, myCAFs, found in close proximity to cancer cells, suggest potential juxtacrine interactions that maintain an immunosuppressive and tumor-supportive microenvironment[49,50]. These insights underscore the complex interplay between DSG2, CSCs, and CAFs in shaping the TME and influencing cancer progression and therapeutic responses. Understanding these interactions holds promise for developing targeted therapies aimed at disrupting these pathways to mitigate tumor aggressiveness and improve patient outcomes.

Our study suggests several avenues for therapeutic intervention targeting the

interactions between DSG2+ CSCs and FAP+ myCAF as well as disrupting the MDK-NCL signaling pathway. Given the role of DSG2 in therapy resistance and as a serum marker for therapy response, monoclonal antibodies targeting DSG2 could be developed to selectively eliminate DSG2+ CSCs, potentially enhancing the effectiveness of existing chemotherapies and targeted therapies. Additionally, small molecule inhibitors designed to disrupt DSG2 function could prevent the maintenance and survival of CSCs, thereby reducing tumor aggressiveness and resistance to treatment. Combining therapies that target DSG2+ CSCs with those that inhibit FAP+ myCAF could disrupt the supportive microenvironment that sustains CSCs. For instance, antifibrotic agents targeting FAP could be used alongside DSG2-targeted therapies to dismantle the protective niche for CSCs. Therapeutic agents that inhibit the physical interaction between DSG2+ CSCs and FAP+ myCAF could also be developed, which might include molecules that interfere with adhesion pathways or disrupt signaling molecules involved in their interaction. As our spatial analysis identified MMP9 and MMP12 expression by FAP+ myCAFs, using MMP inhibitors could prevent the EMT and invasive behavior of CSCs, reducing tumor invasiveness and improving response to therapies.

Our study identified the MDK-NCL signaling pathway as highly active in cell-cell communication at the tumor boundary. Inhibitors targeting MDK or its receptor NCL could be developed to disrupt this pathway, potentially impairing the supportive signaling network for CSCs and enhancing the effectiveness of immunotherapies. Strategies to reduce or block the production of MDK could diminish its signaling activity and impact on CSC maintenance. We propose conducting preclinical studies to evaluate the efficacy of the aforementioned therapeutic strategies in vitro and in vivo, determining the potential of targeting DSG2+ CSCs, FAP+ myCAF, and the MDK-NCL signaling pathway in reducing tumor growth and improving treatment responses. Based on promising preclinical results, early-phase clinical trials could be designed to test the safety and efficacy of these novel therapeutic approaches in patients with cancer types exhibiting high levels of DSG2+ CSCs and FAP+ myCAF.

Several limitations should be considered. Associations between DSG2 expression and treatment response were derived from retrospective analyses of clinical cohorts and require independent and prospective validation. The functional consequences of CSC－myCAF co-localization were inferred from spatial and transcriptomic data and remain to be experimentally confirmed. Moreover, additional orthogonal approaches, such as flow cytometry or independent single-cell platforms, would strengthen validation. Despite these limitations, our study provides a spatial and functional framework for understanding CSC-driven resistance in NSCLC and identifies actionable targets for therapeutic intervention.

Materials and Methods

Patient samples

Formalin-fixed paraffin-embedded (FFPE) specimens were collected from 70 untreated non-small cell lung cancer (NSCLC) patients at the Cancer Hospital, Chinese Academy of Medical Sciences (Beijing, China). All samples were obtained with informed consent and in accordance with institutional ethical guidelines. The study protocol was approved by the Ethics Committee of Institut Curie (Approval No. 23/262-4004). Among these samples, all 70 were used for immunohistochemistry, and six representative cases were further subjected to spatial transcriptomics analysis.

Data sources

Multiple publicly available datasets were integrated to characterize NSCLC at both single-cell and bulk levels. Single-cell RNA sequencing data from GSE148071 (172,164 cells from 57 tumors) were used as the primary dataset, with GSE127465, GSE143423, and EMTAB6149 serving as validation cohorts. Additional single-cell data from 49 samples of 30 patients receiving EGFR-TKI therapy were included to assess treatment-associated changes. Bulk transcriptomic and clinical data were obtained from The Cancer Genome Atlas (TCGA), while mRNA expression profiles from 44 targeted-therapy-treated patients were retrieved from the IMPACT project.

Dimension reduction and cell clustering

Highly variable genes (top 2000) were identified using the FindVariableFeatures function and subjected to principal component analysis. Cell clustering was performed using the FindNeighbors and FindClusters functions. Major cell types were annotated based on canonical markers, including epithelial (EPCAM, KRT8, KRT19), fibroblast (COL1A1, COL1A2, DCN), endothelial (PLVAP, VWF, PECAM1), T cell (CD3D, CD3E, TRAC), B cell (MS4A1, CD79A), and myeloid (CD14, CD163, CD68, FCGR3A) markers.

InferCNV analysis

Large-scale chromosomal copy number variations were inferred using InferCNV. Fibroblasts and endothelial cells were used as reference populations. InferCNV objects were generated from raw count matrices, cell annotations, and gene position files, and analyses were conducted with default settings (cutoff = 0.1, cluster_by_groups = TRUE, denoise = TRUE, HMM = FALSE).

CytoTRACE analysis

Cell differentiation status was estimated using the CytoTRACE algorithm, which assigns each cell a score reflecting transcriptional diversity and stemness. CytoTRACE scores for malignant cells were calculated with the CytoTRACE R package, ranging from 0 to 1, with higher values indicating lower differentiation and increased stem-like features.

Definition of cancer stemness

Given the plastic and reversible nature of cancer stemness, cancer stem cells were not defined as a discrete population. Instead, stemness was treated as a continuous trait across malignant cells, quantified by CytoTRACE scores, where higher scores denote stronger stem-like potential.

Transcription factor activity inference

Transcription factor (TF) activity was inferred using the DoRothEA framework based

on curated TF–target interactions. Human DoRothEA regulons with confidence levels A–C were selected, and regulon activity was estimated using the VIPER algorithm via the run_viper function, integrating expression levels of TFs and their targets.

Metacell analysis

Tumor cells were aggregated into metacells using the R metacell package. Mitochondrial genes were excluded prior to analysis. Genes with scaled variance greater than 0.08 were selected to compute cell–cell similarity. K-nearest neighbor graphs (K = 100) were constructed, followed by 500 resampling iterations using 75% of cells to generate coclustering graphs with a minimum metacell size of 50. Metacell-level gene expression and CytoTRACE scores were calculated by averaging values from constituent cells.

WGCNA

The R package WGCNA was applied to identify gene modules associated with cancer stemness. The pickSoftThreshold function was used to select the optimal soft-thresholding power for network construction. A weighted adjacency matrix was generated, followed by hierarchical clustering based on the topological overlap matrix (1 − TOM) to define gene modules. Module–trait correlations were then calculated against CytoTRACE scores.

AUCell

Gene expression rankings for each cell were first generated using AUCell_buildRankings with default settings. The AUCell_calcAUC function subsequently computed area-under-the-curve (AUC) scores to quantify enrichment of cancer stem cell (CSC)-related gene signatures. Higher AUC values indicated stronger CSC-like transcriptional programs.

Plasma proteomics in patients receiving third-generation EGFR-TKI therapy

Plasma samples from 186 NSCLC patients enrolled in the BPI-7711 phase I (NCT03386955) and phase IIa (NCT03812809) trials were analyzed. All patients had locally advanced or metastatic/recurrent disease harboring EGFR T790M mutations and had progressed on first- or second-generation EGFR-TKI therapy or presented with T790M at diagnosis.

Blood was collected in EDTA tubes and centrifuged at 16,000 g for 10 minutes at 4°C. Isolated plasma was stored at −80°C until analysis. Treatment response was evaluated using Response Evaluation Criteria in Solid Tumours (RECIST v1.1), and patients were classified as responders (complete or partial response) or non-responders (stable or progressive disease). All procedures complied with the Declaration of Helsinki and were approved by the institutional ethics committee.

Survival analysis

Survival analyses were conducted using the survival package in R. Optimal cutoffs for gene expression or cell infiltration were determined using maxstat.test based on maximally selected rank statistics. Kaplan–Meier curves were generated, and group differences were assessed using the log-rank test.

Pathological annotation of spatial transcriptomics samples

Details of spatial transcriptomics sequencing and CNV-based tumor cell identification are provided in the supplementary materials. Each Visium spot was independently evaluated by two pathologists and assigned to histological categories, including normal epithelium, tumor, fibroblast, endothelial, or immune cells. Classification required at least 50% coverage by a given cell type.

Clustering of spatial transcriptomics data

Gene–spot count matrices were processed using Seurat. Data normalization was performed with SCTransform, followed by identification of variable features and clustering using FindVariableFeatures, FindNeighbors, and FindClusters.

CARD deconvolution

Cellular composition of spatial transcriptomics data was estimated using the CARD R package. Single-cell reference profiles for six major cell types were integrated with spatial data using createCARDObject. Deconvolution was performed with CARD_deconvolution, and cell-type proportions were visualized using CARD.visualize.pie.

ORIENT-3 cohort

The ORIENT-3 phase III trial was conducted across 39 centers in China with ethics approval and informed consent from all participants (NCT03150875). Overall survival was the primary endpoint. Patients receiving anti-PD-1/PD-L1 therapy before progression were excluded from the docetaxel arm analysis. Among 157 sequenced patients, 110 had qualified archival FFPE tumor samples with validated RNA sequencing data (61 sintilimab, 49 docetaxel) and were included in downstream analyses. RNA was extracted using the RNeasy FFPE Kit (Qiagen).

Cell–cell communication analysis

Intercellular communication was inferred using CellChat. Signaling interactions were analyzed with CellChatDB.human, and the functional roles of cell populations were assessed using netAnalysis_signalingRole_scatter.

Statistical analysis

Comparisons between two groups were performed using the Mann–Whitney U test. Associations were evaluated using Spearman correlation. All tests were two-sided,

and $P < 0.05$ was considered statistically significant. Data processing and visualization were conducted in R (version 4.1.0).

18% Overall Similarity

Top sources found in the following databases:

- 15% Internet database
- 16% Publications database
- Crossref database
- Crossref Posted Content database

TOP SOURCES

The sources with the highest number of matches within the submission. Overlapping sources will not be displayed.

1	ncbi.nlm.nih.gov<br>Internet	2%
2	assets.researchsquare.com<br>Internet	2%
3	cslide.ctimeetingtech.com<br>Internet	1%
4	journals.lww.com<br>Internet	<1%
5	frontiersin.org<br>Internet	<1%
6	Guangyu Fan, Ruyun Gao, Tongji Xie, Lin Li, Le Tang, Xiaohong Han, Yu...<br>Crossref	<1%
7	Guangyu Fan, Tongji Xie, Lin Li, Le Tang, Xiaohong Han, Yuankai Shi. "S...<br>Crossref	<1%
8	Guangyu Fan, Tongji Xie, Qiaoyun Tan, Ning Lou, Shasha Wang, Xiaoho...<br>Crossref	<1%
9	Tiantian Du, Huiru Gao, Honglei Wu, Juan Li et al. "Comprehensive diss...<br>Crossref posted content	<1%

10	pmc.ncbi.nlm.nih.gov<br>Internet	<1%
11	medrxiv.org<br>Internet	<1%
12	translational-medicine.biomedcentral.com<br>Internet	<1%
13	NA. "Supplement 2, Proceedings of the 14th World Conference on Lun...<br>Crossref	<1%
14	Tianyu Zeng, Yang Wang, Bo Tang, Haipeng Cui et al. "Colorectal Liver ...<br>Crossref	<1%
15	worldwidescience.org<br>Internet	<1%
16	jeb.biologists.org<br>Internet	<1%
17	pubmed.ncbi.nlm.nih.gov<br>Internet	<1%
18	biorxiv.org<br>Internet	<1%
19	preprints.org<br>Internet	<1%
20	Jiaxian Ong, Sharanya Sritharan, Amar Harris Bin Arifin, Baskaran Gun...<br>Crossref	<1%
21	Youjie Zeng, Si Cao, Minghua Chen. "Integrated analysis and exploratio...<br>Crossref	<1%

22	Mona P. Roshan, Grayson V. Gigliotti, Jeffrey Gonzalez, Ricardo A. Cur... Crossref	<1%
23	jcancer.org Internet	<1%
24	oatext.com Internet	<1%
25	pure.solent.ac.uk Internet	<1%
26	science.gov Internet	<1%
27	Benjamin Siciliano, Nicholas D. Henkel, William G. Ryan V, Ali Sajid Ima... Crossref	<1%
28	ddd.uab.cat Internet	<1%
29	Jiani Wu, Dongqiang Zeng, Shimeng Zhi, Zilan Ye et al. "Single-cell anal... Crossref	<1%
30	Parteek Prasher, Mousmee Sharma, Indu Pal Kaur, Ronan MacLoughlin... Publication	<1%
31	elifesciences.org Internet	<1%
32	public-pages-files-2025.frontiersin.org Internet	<1%
33	emjreviews.com Internet	<1%

34	"Annual Congress of the European Association of Nuclear Medicine Oc...<br>Crossref	<1%
35	Rojas, Ernesto Javier. "Developmental Origins and Molecular Mechanis...<br>Publication	<1%
36	William E. Pierceall, Steven L. Warner, Ryan J. Lena, Camille Doykan et ...<br>Crossref	<1%
37	bioone.org<br>Internet	<1%
38	Guangyu Fan, Changcheng Tao, Lin Li, Tongji Xie, Le Tang, Xiaohong H...<br>Crossref	<1%
39	Wenchao Xia, Tan Lin, Mengnan Shi, Qiuqiao Mu, Han Zhang, Yijun Xu. ...<br>Crossref	<1%
